# Polymorphic *Cis*- and *Trans*-Regulation of Human Gene Expression

**DOI:** 10.1371/journal.pbio.1000480

**Published:** 2010-09-14

**Authors:** Vivian G. Cheung, Renuka R. Nayak, Isabel Xiaorong Wang, Susannah Elwyn, Sarah M. Cousins, Michael Morley, Richard S. Spielman

**Affiliations:** 1 Howard Hughes Medical Institute, Philadelphia, Pennsylvania, United States of America; 2 Department of Pediatrics, University of Pennsylvania, The Children's Hospital of Philadelphia, Philadelphia, Pennsylvania, United States of America; 3 Department of Genetics, University of Pennsylvania, The Children's Hospital of Philadelphia, Philadelphia, Pennsylvania, United States of America; 4 University of Pennsylvania, The Children's Hospital of Philadelphia, Philadelphia, Pennsylvania, United States of America; 5 Medical Scientist Training Program, University of Pennsylvania, The Children's Hospital of Philadelphia, Philadelphia, Pennsylvania, United States of America; The Wellcome Trust Centre for Human Genetics, University of Oxford, United Kingdom

## Abstract

Expression levels of human genes vary extensively among individuals. This variation facilitates analyses of expression levels as quantitative phenotypes in genetic studies where the entire genome can be scanned for regulators without prior knowledge of the regulatory mechanisms, thus enabling the identification of unknown regulatory relationships. Here, we carried out such genetic analyses with a large sample size and identified *cis*- and *trans*-acting polymorphic regulators for about 1,000 human genes. We validated the *cis*-acting regulators by demonstrating differential allelic expression with sequencing of transcriptomes (RNA-Seq) and the *trans*-regulators by gene knockdown, metabolic assays, and chromosome conformation capture analysis. The majority of the regulators act in *trans* to the target (regulated) genes. Most of these *trans*-regulators were not known to play a role in gene expression regulation. The identification of these regulators enabled the characterization of polymorphic regulation of human gene expression at a resolution that was unattainable in the past.

## Abstract 2

Using genetic and molecular analyses, we identified over 1,000 polymorphic regulators that regulate expression levels of human genes.

## Author Summary

Cellular characteristics and functions are determined largely by gene expression and expression levels differ among individuals, however it is not clear how these levels are regulated. While many *cis*-acting DNA sequence variants in promoters and enhancers that influence gene expression have been identified, only a few polymorphic *trans*-regulators of human genes are known. Here, we used human B-cells from individuals belonging to large families and identified polymorphic *trans*-regulators for about 1,000 human genes. We validated these results by gene knockdown, metabolic perturbation studies and chromosome conformation capture assays. Although these regulatory relationships were identified in cultured B-cells, we show that some of the relationships were also found in primary fibroblasts. The large number of regulators allowed us to better understand gene expression regulation, to uncover new gene functions, and to identify their roles in disease processes. This study shows that genetic variation is a powerful tool not only for gene mapping but also to study gene interaction and regulation.

## Introduction

Expression levels of genes, like many phenotypes, vary among normal individuals. Since gene expression underlies cellular characteristics and functions, variation in gene expression contributes to phenotypic diversity and differences in disease susceptibility. Previously, we and others demonstrated that there is a genetic basis to individual variation in gene expression [1]–[6]. This facilitates studies to identify sequence variants that influence expression levels of genes. Since expression phenotypes of many genes are studied in parallel, results from genetics of gene expression (GOGE) studies contribute to the understanding of global gene regulation.

GOGE studies that treated expression levels as quantitative traits in family-based linkage [3],[7] and population-based association analyses [5],[8],[9] have uncovered polymorphic regulatory regions that contribute to variation in human gene expression. However, the regulatory regions were large, often megabases in size; thus, the identity of most polymorphic regulators remained unknown. In this GOGE study, we analyzed a large sample in linkage analyses, then we used deep sequencing of transcriptomes (RNA-Seq) to guide association-based fine mapping. The results allowed us to narrow the regulatory regions and identify *cis*- and *trans*-acting polymorphic regulators of ∼1,000 human genes. These results facilitated molecular validation and analyses of the mapping data. This is an important advance in human genetic studies where such validations have largely been impossible. In previous human GOGE studies, the resolution of the mapping results was inadequate; hence, regulators were not identified, while other gene mapping studies focused on complex phenotypes, such as human diseases that are often not amenable to molecular analyses. Thus, the end points of many human genetic studies showed genotype-phenotype connections statistically but not molecularly.

Here, we have an unusual opportunity to begin to bridge the gap between genetic and mechanistic studies. Knowing the identity of the regulators, we were able to validate the *cis*- and the *trans*-regulatory relationships using different approaches. For genes that are *cis*-regulated, we used RNA-Seq to show differential allelic expression. For the *trans*-regulatory relationships, we altered the expression of the regulators by gene knockdowns and metabolic perturbations and showed that manipulations of the regulators affected the expression levels of the corresponding target genes. We also demonstrated direct interactions between regulators and their target genes by chromosome conformation capture.

Another goal of this study is to examine the role of *cis*- and *trans*-acting polymorphisms on human gene expression. Previously, GOGE studies in model organisms and humans appear to disagree on the proportion of polymorphic *cis*- and *trans*-acting regulators. In yeast, fly, and mouse studies, most of the regulators act in *trans* [2],[10]–[12]. In contrast, human studies focused mostly on *cis*-acting variants. This apparent discrepancy is likely due to differences in sample sizes. Studies in model organisms used larger sample sizes and thus were able to find *trans*-acting regulators that have smaller effects on gene expression than *cis*-regulators [13],[14]. In contrast, early human studies of GOGE used relatively small sample sizes, such as samples collected by the International HapMap Consortium [5],[8]; hence they identified mostly *cis*-regulators. This and the discovery of *cis*-regulation of disease susceptibility genes such as *ORMDL3* (asthma) [15] led to suggestions that *cis*-acting variants are significant contributors to variation in human gene expression. However, it is unlikely that the regulatory landscapes are different between humans and other organisms. In humans, *trans*-acting regulators possibly also play an important role. Several studies [3],[4],[9] have suggestive evidence for the important contribution of *trans*-acting variants. Recently, studies that used RNA-Seq to analyze gene expression phenotypes in HapMap samples found *cis*-acting variants for less than 10% of human genes [16],[17]. These studies suggest that along with *cis*-variants, *trans*-acting polymorphisms contribute to individual variation in human gene expression. Here, to address this, we used a large sample size and identified hundreds of polymorphic *trans*-regulators. These findings confirm that as in other organisms, there are many sequence variants in the human genome that act in *trans* to influence gene expression.

Many of the identified *trans*-regulators were previously not known to play a role in gene regulation. Over 60% of the regulators are not transcription factors or known signaling factors. However, the *trans*-regulators are not randomly distributed; instead they tend to be found in the same functional pathways as their target genes. While the regulators were discovered in analysis of immortalized B-cells, we showed that the regulatory relationships were also found in primary fibroblasts. Thus, natural variation in gene expression allowed the identification of polymorphic expression regulators, which then enabled us to develop a deeper understanding of gene regulation.

## Results

### Linkage Scans

**Figure 1:**
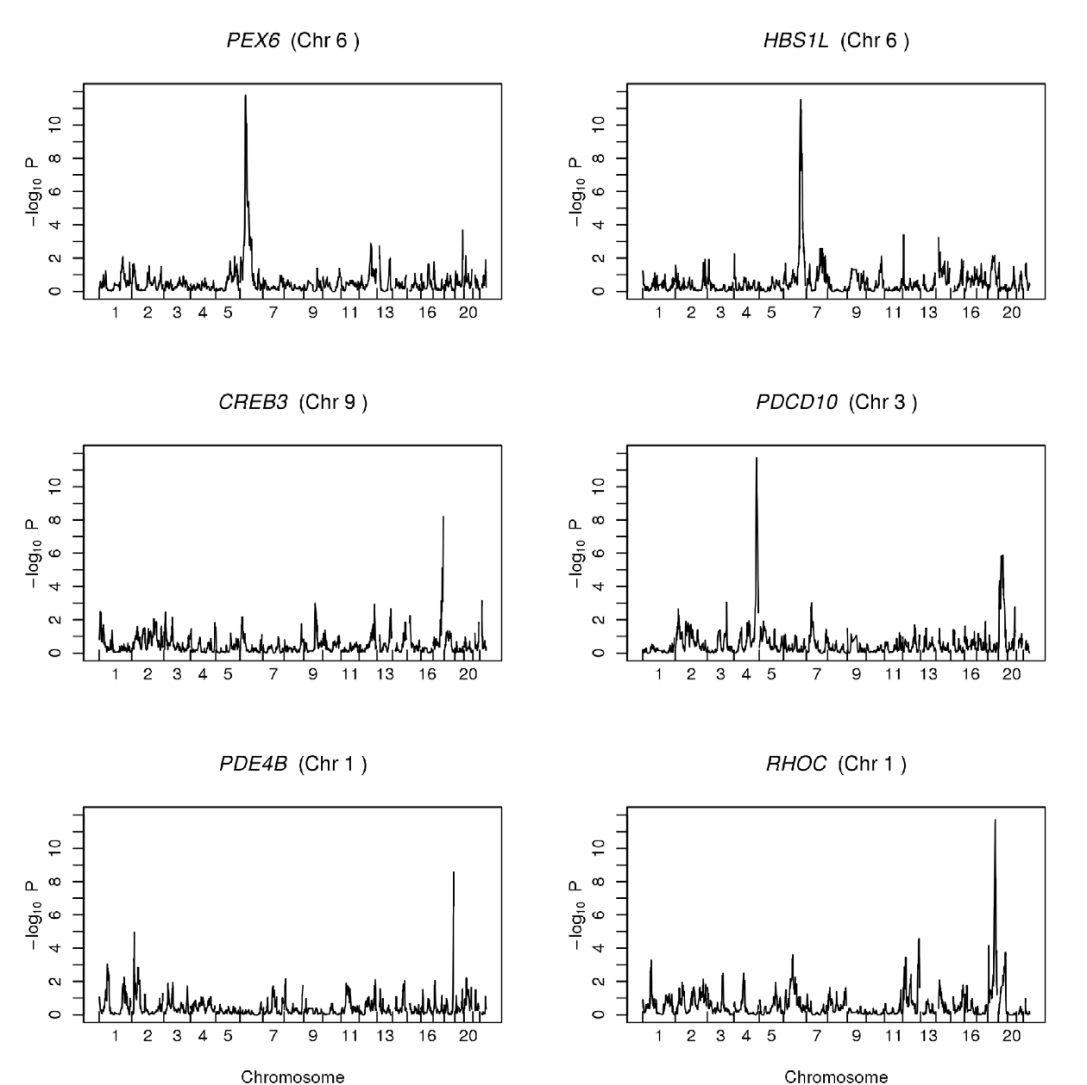
Genome scans of six expression phenotypes. The name of the target gene and its chromosomal location (in parenthesis) are shown. Evidence of linkage as indicated by *p* value (−log_10_) is shown on the vertical axis and genomic locations are shown on the horizontal axis of each graph. The top two panels are examples of phenotypes with proximal linkage peaks, and the bottom four panels are phenotypes with distal linkage peaks.

We obtained genotypes of single nucleotide polymorphisms (SNPs) and measured the expression levels of genes in immortalized B-cells from members of 45 Centre d'Etude du Polymorphisme Humain (CEPH) Utah pedigrees [18] using microarrays. We focused our analysis on 4,793 expressed genes that show variation in expression levels among individuals and carried out genome-wide linkage analysis (see Methods). From those analyses, we selected 1,681 (35%) phenotypes for further studies using a threshold of *t*>4 (a logarithm of odds (lod) score of ∼3.4, and a genome-wide corrected significance level of approximately 0.05 [19]) (see Methods). Figure 1 shows examples of genome scan results.

We expected to find polymorphic regulators of the expression phenotypes in the candidate regions identified by the linkage scans. Hence we examined the linkage peaks to determine their locations relative to the genomic addresses of the target genes. To take into account the imprecision of linkage, we define regulatory regions that are within 5 Mb of the target genes as proximal and those that are greater than 5 Mb or on another chromosome as distal to the target genes [20]. By this definition, among the 1,681 phenotypes with evidence of linkage at *t*>4, we found that 70 (4.2%) phenotypes have proximal regulators, 1,574 (93.6%) phenotypes have distal regulators, and 37 (2.2%) phenotypes have both proximal and distal regulators. Ninety-four percent of the distal regulators are on a different chromosome than their corresponding target genes. These results suggest that *trans*-acting regulation contributes appreciably to variation in gene expression.

### Family-Based and Population Association

Linkage scans provided regulatory regions for over 1,600 expression phenotypes. To confirm these results, we carried out family-based and population-based association analyses with markers within the candidate regulatory regions. In addition to confirming the linkage findings, association mapping allows us to take advantage of historical recombinations in order to narrow the candidate regions.

#### Proximal linkage peaks

For the 107 (70+37) phenotypes where the linkage peaks are proximal to the target genes, we assumed that they are likely to be *cis*-regulated, so we tested SNPs within and 50 kb up- and downstream of the target genes. Among these 107 phenotypes, we had informative genotypes for 100 phenotypes to carry out family-based association analysis by quantitative transmission disequilibrium test (QTDT) [21]. From the analysis of the members of the 45 CEPH pedigrees by QTDT, 63 of the 100 phenotypes showed significant evidence (nominal *p*≤0.001) for the combined presence of linkage and association (Table S1). These results confirm the linkage findings and support that these phenotypes are *cis*-regulated.

Using expression data and genotypes of 86 unrelated individuals, we carried out population-based association analysis and found significant evidence (nominal *p*<0.005) for population associations between gene expression levels and SNPs within or near the target genes (Table S1) for 47 (75%) of these 63 phenotypes. We also estimated the variation in expression explained by the *cis*-acting determinants by calculating *R* ^2^ using results of the linear regression analyses in population association studies. For the 47 phenotypes, the average *R* ^2^ is 0.25 (range = 0.09 to 0.75). For 17 of these phenotypes, the *cis*-variants explained more than 30% of the individual variation in their expression levels. This provides an estimate of the contribution of *cis*-variants; the fraction not explained this way includes non-genetic factors (including environment) and other genetic factors not in linkage disequilibrium with the *cis*-acting determinants.

**Figure 2:**
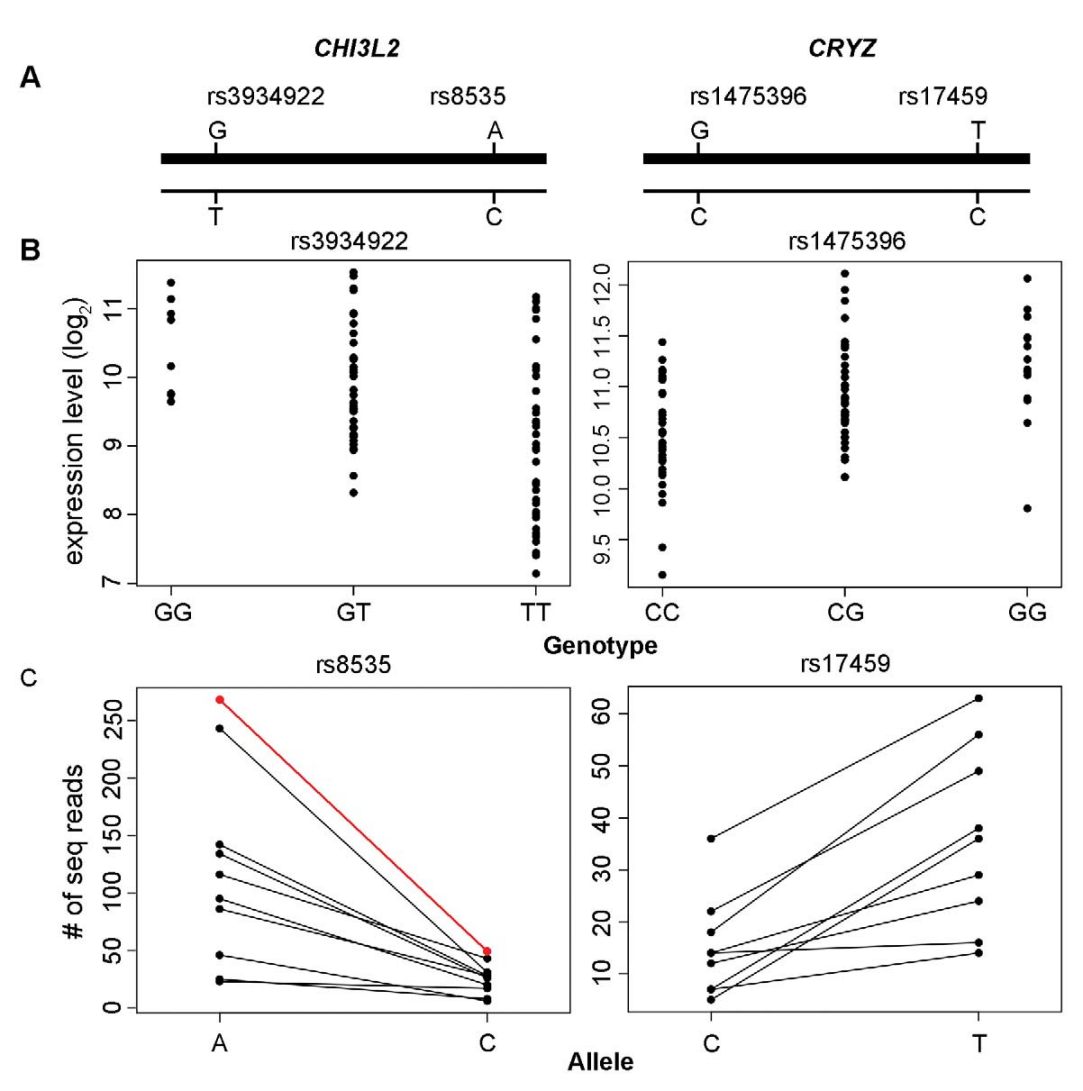
Allelic expression from RNA-Seq confirms prediction by association analysis. Graphical presentations of two genes that show differential allelic expression. The thick lines represent the higher expressing allelic forms of *CHI3L2* and *CRYZ* (A). Regression of expression phenotypes (expression levels shown on vertical axis) of two genes on nearby SNPs (genotypes shown on horizontal axis) (B). Number of reads (vertical axis) from RNA-Seq for each allelic form (horizontal axis) of the genes; only data for individuals who are heterozygous at the coding SNPs are shown. For each individual, the number of reads for each allele of an SNP is connected by a line. For example, in the panel for rs8535 (*CHI3L2*), the individual represented by a red line had 268 reads of the A-bearing form of *CHI3L2* and 49 reads of the C-bearing form of *CHI3L2* (C).

These findings further support the linkage results and provide evidence of differential allelic expression of these genes (see Figure 2 for examples). We also looked for molecular evidence of *cis*-regulation (see section below on differential allelic expression under “molecular validation”).

#### Distal linkage peaks

We followed up results for the 1,611 (1,574+37) phenotypes with significant distal linkage peaks using QTDT. Unlike proximal peaks where we can look for *cis*-acting variants within or near the target genes, there are no obvious regions to look in the distal peaks. This is particularly difficult when the linkage peaks are large and contain several potential regulators. One option is to test all the SNPs under the linkage peaks for evidence of association with expression levels of the corresponding target genes; however, that would result in a severe multiple testing problem. Instead we identified the genes that are expressed in our B-cells by RNA-sequencing and testing the expressed genes for association with expression levels of their corresponding target genes.

In RNA-sequencing, we are not limited to studying only genes that are represented on the microarrays. With sufficient coverage, sequencing data also allow us to detect genes that are expressed at lower levels. This is important since gene expression regulators, such as transcription factors, are often expressed at low levels. We sequenced the cDNA samples of 41 CEPH HapMap (CEU) [22],[23] individuals using the Illumina technology [24]. For each sample, we obtained ∼40 million reads, each 50 nucleotides long or about 2 Gb of sequences per sample. We mapped the short read sequences to the reference human genome (hg18) using the software MAQ [25]. About 83% of the sequences mapped uniquely to the reference sequence. The other 17% mapped to multiple sites in the genome (repetitive sequences or sequence motifs that are common in gene families) or failed to map anywhere in the reference genome (including exon junctions which would not map to the reference genome sequence). We compared the expression levels of genes in B-cells of the same individuals from RNA-Seq with those from our microarrays, the average correlation was 0.76 (range = 0.73 to 0.80, highly similar to those in other studies [16],[17],[26]). We also compared genotypes from our RNA-Seq to those from the HapMap Consortium and found the average concordance rate was 98.6%. These results gave us confidence in the data so we used them to identify expressed genes in the candidate regions identified by linkage scans.

We tested SNPs in the expressed genes within each linkage peak for association with their candidate target genes. For genes that are expressed with RPKM≥1 [26] in the linkage regions, we carried out QTDT analyses using SNPs within and 5 kb up- and downstream of the regulators in all members of the 45 CEPH families. Among the 1,611 phenotypes with distal linkage peaks, we excluded 94 phenotypes whose candidate regulatory regions were over 20 megabases in size. Of the 1,517 remaining phenotypes, the expression levels of 103 (6.8%) phenotypes showed evidence of linkage and association (nominal *p*≤0.001; FDR = 0.02) with SNPs in *trans*-regulators. Since *trans*-acting regulators have weaker effect than *cis*-regulators, we also looked at results using lower thresholds of *p*<0.01 and 0.05 (FDR 3 and 8%); there are 518 (34%) and 917 (60%) phenotypes that met these thresholds, respectively. Among these 917 phenotypes, the expression levels of 112 genes are influenced by two or more unlinked polymorphic *trans*-regulators. Thus, the analysis revealed 1,036 regulator–target gene pairs. Table 1 shows the top 20 *trans*-regulator–target gene pairs from our linkage and association study (see 2 for the top 200 regulator–target gene pairs). As we did for the *cis*-acting regulators, we tested the *trans*-regulators identified by QTDT for allelic association with their target genes using population association tests. Although the sample size is small (*n* = 86), we found significant association for a set of *trans*-regulators and their target genes. Among the regulators of the 917 target genes, SNPs in 58 (6%) and 318 (35%) regulators showed significant allelic association with expression levels of their target genes at nominal *p*<0.005 and *p*<0.05, respectively. To estimate the influence of these *trans*-acting variants on individual variation in gene expression, we calculated *R* ^2^ for the 318 phenotypes. The average *R* ^2^ is 0.07 (median = 0.07; range = 0.05 to 0.24). For 53 phenotypes, the *trans*-acting variants explained 10% or more of the individual variation in their expression levels. These results show that individual differences in gene expression can be explained by DNA sequence polymorphisms in *trans*-acting regulators.

**Table 1:** Expression phenotypes with the strongest evidence of linkage and association to polymorphic *trans*-regulators.

Target Gene	Target Gene (Chr)	*t* Value^1^	*Trans*-Regulator	Regulator (Chr)	SNP (QTDT)^2^	*p* Value (QTDT)	SNP (Association)	*p* Value (Association)^3^	Expression Level (Log2) by Genotype
*PECAM1*	17	5.54	*PSMD8*	19	rs2074981	2×10^−5^	rs2074981	0.02	(4.62, 7.22, 6.85), (“AA,” “AC,” “CC”)
*DLG5*	10	5.21	*TNIK*	3	rs9814699	2×10^−5^	rs9810370	0.03	(5.47, 5.93, 6.15), (“AA,” “TA,” “TT”)
*TYMS*	18	4.96	*OPTN*	10	rs17512962	2×10^−5^	rs2095387	N.S.	(13.02, 12.9, 13.04), (“GG,” “TG,” “TT”)
*SSR1*	6	4.58	*ITPR2*	12	rs12823128	2×10^−5^	rs12823128	0.002	(11.01, 10.97, 10.69), (“CC,” “TC,” “TT”)
*FOXG1*	14	4.44	*NAPB*	20	rs2424534	2×10^−5^	rs2252824	0.04	(3.83, 3.12, 4.41), (“AA,” “AG,” “GG”)
*PARVA*	11	4.23	*VGLL4*	3	rs6807423	2×10^−5^	rs12374138	0.005	(6.02, 5.78, 5.23), (“AA,” “AG,” “GG”)
*ATXN2L*	16	4.21	*ENOPH1*	4	rs1980187	2×10^−5^	rs6826022	N.S.	(7.34, 7.32, 7.1), (“CC,” “CT,” “TT”)
*PDE4B*	1	5.89	*MBP*	18	rs9959822	3×10^−5^	rs11150996	0.02	(8.36, 8.06, 7.92), (“CC,” “CT,” “TT”)
*USP1*	1	4.05	*UXS1*	2	rs17279736	3×10^−5^	rs2167531	N.S.	(10.65, 10.7, 10.83), (“CC,” “CT,” “TT”)
*KHDRBS3*	8	4.72	*FAM120B*	6	rs910424	4×10^−5^	rs1022615	0.002	(4, 4.92, 6.22), (“CC,” “GC,” “GG”)
*ZNF189*	9	4.85	*ARHGAP10*	4	rs6822971	8×10^−5^	rs7660368	N.S.	(8, 8.03, 8.27), (“CC,” “TC,” “TT”)
*USPL1*	13	4.68	*COG1*	17	rs1026129	9×10^−5^	rs1026128,	N.S.	(8.32, 8.33, 8.34), (“AA,” “AG,” “GG”),
*IFNA2*	9	4.81	*C3orf1*	3	rs1967621	1×10^−4^	rs1967621	N.S.	(4.29, 4.61, 4.68), (“CC,” “GC,” “GG”)
*PDCD10*	3	4.72	*ZNF429*	19	rs2650825	1×10^−4^	rs2650825	N.S.	(10.66, 10.75, 10.83), (“CC,” “CT,” “TT”)
*MLYCD*	16	4.64	*DNAJC25-GNG10*	9	rs1322251	1×10^−4^	rs10817199	N.S.	(7.66, 7.65, 7.27), (“CC,” “GC,” “GG”)
*KCNMB3*	3	4.57	*IPO8*	12	rs3910561	1×10^−4^	rs33270	0.04	(5.93, 5.43), (“AA,” “GA”)
*STXBP3*	1	4.38	*DCUN1D2*	13	rs3814254	1×10^−4^	rs2261120	N.S.	(7.5, 7.54, 8.07), (“CC,” “TC,” “TT”)
*ATXN2*	12	4.86	*NFATC2*	20	rs4811172	2×10^−4^	rs6067803	0.03	(8.11, 8.18, 8.4), (“GG,” “GT,” “TT”)
*LIN7A*	12	4.49	*YLPM1*	14	rs2241275	2×10^−4^	rs957345	N.S.	(7.52, 6.89, 6.83), (“CC,” “CG,” “GG”)
*ETV6*	12	5.04	*KCNQ5*	6	rs16883476	2×10^−4^	rs16882712	N.S.	(7.92, 7.41, 7.76), (“AA,” “AG,” “GG”)

1 From linkage scans (S.A.G.E./sibpal) of 45 families (>1,000 sibpairs).

2 QTDT of all members of 45 families.

3 Population association of 86 unrelated individuals.

N.S. = not significant (*p*>0.05). The sample size for population association is much smaller than ones for the linkage and QTDT analyses.

### Known and Newly Discovered Regulatory Relationships

To check the validity of these findings, we looked for known regulatory relationships among the regulator–target gene pairs that we identified in the genetic analyses. An example of such known relationship is *MRLC2*, which encodes myosin regulatory light chain 2 and its regulator myocyte enhancing factor 2A, *MEF2A*, a transcription factor that is known to affect muscle gene expression, including *MRLC2* [27]. Our linkage results identified chromosome 15q26 (linkage *t* = 4.9) as the candidate regulatory region for the expression level of *MRLC2*. Using association analyses, we narrowed the candidate region and rediscovered *MEF2A* as the regulator of expression level of *MRLC2* (QTDT *p* = 0.008; population association *p* = 0.04, rs325380). Another example is TTC5 as the polymorphic regulator of *HSP90AA1* expression. Previous studies showed that a mouse protein phosphatase that contains a tetratricopeptide repeat regulates heat shock protein 90; this regulation occurs by dephosphorylation, which is mediated by the binding of heat shock protein 90 to the tetratricorepeat domain of the phosphatase [28],[29]. Our results showed that the expression of human *HSP90AA1* is influenced by variants in *TTC5*, a gene with a tetratricopeptide repeat (linkage *t* = 5.4; QTDT *p* = 0.01, rs11623837). The “rediscovery” of these known regulatory relationships confirms that our approach can identify *trans*-acting regulators of human gene expression.

For the 20 regulator–target gene pairs in Table 1, we checked for co-occurrence of the names of the regulators and target genes in the literature using a text-mining program, Chilibot [30], to determine if any of these regulatory relationships are known. We also queried PubMed for such co-occurrences. Among these 20 pairs, only one pair (*MBP* and *PDE4B*) has been shown to have interactive relationships in Chilibot. Thus, many of these regulator-target relationships are likely unknown previously.

### Molecular Validation

#### RNA-Seq to identify differential allelic expression in *cis*-regulated genes

To validate the polymorphic *cis*-regulation identified in our mapping study, we used the RNA-Seq data of individuals in the HapMap Project as described above. We used the sequencing data to assess differential allelic expression (DAE) [16],[17],[22],[23].

The digital nature of the sequence data allows us to use the heterozygous genotypes in each transcript to determine whether two allelic forms of a transcript are expressed in equal abundance [31]–[33]. Among the 107 expression phenotypes with proximal linkage peaks, 67 have at least one SNP where there are 2 individuals who are heterozygous at that SNP (see Methods). We examined these heterozygous samples for evidence of DAE. For many of these genes, we have data for multiple SNPs from an average of 7.2 individuals (median = 6). Among the 67 genes, 43 genes (64%) showed significant evidence (*p*<0.01, chi-square test) of departure from equal expression of the two allelic forms of the genes. For the 273 exonic SNPs in these 43 genes, we calculated an “allelic expression ratio” a/(a+b), where a and b are the numbers of sequence reads for the two alleles. Figure S1 shows these allelic expression ratios and their departures from 0.5. For 31 of these genes, the exonic and the associated SNPs from our mapping study are part of the markers in the HapMap Project [23]; thus phased haplotype data are available. Using these haplotypes, we showed that for 28 (90%) genes, the predicted expression in the association study was confirmed by RNA-Seq data (see Figure 2 for examples). Hence, the DAE results confirm the findings from our mapping studies and show that the majority of genes (∼65%) with proximal linkages are *cis*-regulated. For the remaining phenotypes, either we do not have adequate sample size or read coverage to detect subtle evidence of DAE, or they are regulated by *trans*-regulators that mapped close to the target genes.

Our sequence data allow us to examine DAE of many more genes in addition to those with proximal linkage peaks. There are 5,782 genes that can be studied for DAE. Among them, 1,029 (18%) and 1,501 (26%) genes showed significant evidence of DAE at *p* value (chi-square test) thresholds of 0.001 and 0.01, respectively. The 18% to 26% of genes that show DAE provide another estimate of the number of genes in our B-cells that are *cis*-regulated. This proportion is similar to the 12% estimated by Price and colleagues using admixture analysis [34], the 30% by Pastinen and colleagues by hybridization of cDNA to SNP arrays [35], the 11% to 22% by Church and colleagues using RNA-Seq, and our mapping study (we found 6.5% and 24% of phenotypes to have proximal peaks, at *t* threshold of 4 and 5, respectively).

#### Molecular validation of *trans*-acting regulators: gene knockdown

To validate the *trans*-regulator–target gene relationships, we carried out molecular analyses. First, we performed gene knockdown studies. We used short interfering RNA (siRNA) to silence 25 potential regulators, and then assessed the effects by measuring the expression of the target genes (we tested cells from 4 to 6 individuals) [36]. Among the 25 regulators, we included *MEF2A* and *TTC5* as positive controls. The remaining 23 regulators were selected based on availability of siRNAs and they span a range of QTDT significance from *p* = 10^−5^ to 10^−2^ (for *BLM*-*NUSAP1*). We did not select regulators that were supported by the most significant *p*-values. We reasoned that if we can confirm molecularly the regulators with relatively modest statistical supports, then the ones with more significant mapping results are likely to be true regulators.

Among the 25 regulators, successful knockdown was achieved for 18 regulators. The expression of these regulators including *MEF2A* and *TTC5* decreased significantly (*p*<0.05) by about 20% to 85% in four or more independent samples, whereas no changes in the expression of the regulators were observed when siRNAs with no homology to the regulators were used (Table 2). We then measured the expression levels of the target genes following knockdown of their regulators. The expression levels of 13 (72%) target genes including *MRLC2* (target of MEF2A) and *HSP90AA1* (target of TTC5) changed significantly (*p*<0.05) following the knockdown of their regulators (Table 2). The expression levels of the target genes, such as *NUSAP1* (encodes a spindle associated protein) and *SSR1* (signal sequence receptor alpha), changed by ∼10% to 60%, while those of non-target control (*GAPDH*) did not change significantly after the knockdown of the regulators, suggesting that the changes in expression levels of the target genes were specific effects of silencing their regulators.

**Table 2:** Results of knockdown of *trans*-regulators.

		Changes in Expression Levels of	
Regulator–Target Gene*	Regulator†	Target Gene†	Control (*GAPDH*)‡
*AIG1-TMEM50A*	−51.0±7.8	17.1±2.9	−0.4±13.2
*BLM*-*NUSAP1*	−39.2±7.7	23.3±3.7	0.1±3.3
*CLTA*-*GCA*	−84.2±6.3	22.2±5.3	14.5±1.5
*FAM120B-KHDRBS3*	−71.4±1.2	26.5±1.2	5.8±7.2
*GALNTL4-PTPRG*	−77.5±4.1	36.7±11.8	−3.7±11.1
*GPHN*-*RALB*	−72.6±4.6	40.7±8.7	14.7±12.0
*HSP90AB1*-*STK24*	−79.0±4.7	14.4±5.3	9.2±4.7
*ITGB4BP*-*SLC25A11*	−64.4±4.3	65.7±7.8	0.2±0.4
*ITPR2*-*SSR1*	−21.4±8.5	20.1±6.1	7.1±11.6
*MEF2A-MRLC2*	−33.9±9.3	−28.1±2.6	12.7±8.5
*PSAP*-*HMGCS1*	−68.5±5.1	11.9±6.2	2.7±4
*TTC5-HSP90AA1*	−87.3±3.1	−43.8±12.8	−2.6±14.9
*VGLL4-PARVA*	−65.9±7.3	56.5±14.2	15.2±7.9

*All experiments were based on independent siRNA knockdown of four or more samples.

**†:** Expression levels of the regulators and target genes changed significantly (*p*<0.05, *t* test) compared to baseline (without siRNA knockdown). Results are shown as mean ± S.E.M.

**‡:** Expression level of a control, *GAPDH*, did not change significantly (*p*>0.05) upon siRNA knockdown of the regulators. Results are shown as mean ± S.E.M.

We followed up three of these regulator–target gene pairs in primary fibroblasts (*n* = 2) to determine the cell-type specificity of the regulatory relationships. We carried out siRNA-mediated knockdown of *BLM*, *ITGB4BP*, and *PSAP* in fibroblasts. Following the silencing of *BLM* and *ITGB4BP*, we observed significant changes (*p*<0.05, *t* test) in expression of their target genes *NUSAP1* and *SLC25A11* as in the immortalized B-cells (Table S3A). However, the expression of *HMGCS1* did not change significantly following knockdown of its regulator, *PSAP*, suggesting that this regulatory relationship may be specific for B-cells.

These results provide molecular support for the regulator–target gene relationships identified in our mapping studies. However, the lack of changes in expression levels of the target genes following the knockdown of their regulators does not argue against the regulatory relationships. The expressions of the regulators were only partly decreased by siRNAs; partial expression may be sufficient for regulation. In addition, many human genes have other family members that take over their functions upon knockdown of their expression.

#### *INSR* and its target genes

**Figure 3:**
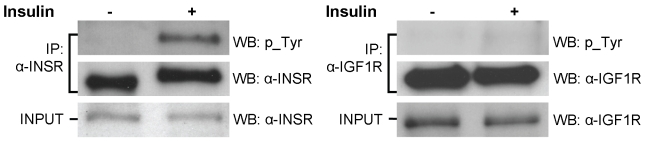
INSR activation following insulin treatment in human primary fibroblasts. Fibroblasts were serum starved for 18 h and then treated with 100 nM insulin for 5 min. Cell lysates were incubated with α-INSR or α-IGF1R antibodies. Input and immunoprecipitated products were analyzed by western blots using α-phosphotyrosine, α-INSR, or α-IGF1R antibodies.

In addition to knockdown studies, we carried out another functional analysis that does not rely on transfection. One of the *trans*-regulators is the insulin receptor, INSR. Our mapping results identified six genes, *ADD3*, *ARNT*, *ATIC*, *CCL5*, *LTB*, and *PSMD10*, whose expression levels are regulated by the insulin receptor, INSR. Previously, studies have shown that *ADD3* is involved in insulin receptor signaling [37]. In addition, Kahn and colleagues showed that following knockdown of *ARNT*, the expression of the insulin receptor was decreased in pancreatic islet cells [38]. If our mapping results are correct, then it would suggest that insulin receptor regulates the expression of *ARNT*, providing evidence of reciprocal regulation of *ARNT* and *INSR* or feedback mechanisms. To validate these regulatory relationships, we stimulated the insulin receptor by treating primary fibroblasts with insulin and measured the expression levels of *INSR* and its target genes. The fibroblasts allowed us to confirm the regulatory relationship in primary cells. Among the six genes, four (*ADD3*, *ARNT*, *ATIC*, and *PSMD10*) were expressed in fibroblasts, so we focused our analysis on these genes. We treated the cells with insulin; upon insulin treatment, the insulin receptor is activated but not the related IGF1 receptor, thus indicating that insulin was acting specifically through INSR (Figure 3). Insulin led to a biphasic response of *INSR*: 2 h after insulin treatment, the expression level of *INSR* among four individuals increased by an average of 12% (*p* = 4×10^−5^); this was followed by a 48% decrease (*p* = 0.004) at 6 h after exposure to insulin (Table 3). The expression levels of the four target genes also changed significantly (*p*<0.005) following insulin treatment (Table 3). These findings confirm our mapping results, which identified *INSR* as the polymorphic regulator of expression of *ADD3*, *ARNT*, *ATIC*, and *PSMD10*. They show that the regulatory relationships identified in immortalized B-cells are also found in primary fibroblasts.

**Table 3:** Changes in expression levels of insulin receptor target genes following insulin treatment.

	Time Point Following Insulin Treatment			
Gene Names	1 h	2 h	6 h	12 h
*INSR*	11%	12%*	−48%*	2%
*ARNT*	−3%*	−9%*	−39%*	−16%*
*ATIC*	−2%*	3%*	49%*	82%*
*ADD3*	7%*	10%*	−19%*	−37%*
*PSMD10*	13%	12%*	−8%	13%*

*p<0.005 compared to no treatment (*t* test, *n* = 4).

#### Physical interactions between regulators and their target genes

We used data from chromosome conformation capture (3C) as additional validation of the regulatory relationships and to identify regulatory pairs that interact physically. Recently, using the same immortalized B-cells, Dekker and colleagues combined 3C with high-throughput sequencing, in a procedure they coined “Hi-C,” to identify regions of the human genome that interact physically [39]. We used their data to determine if any of our 1,036 regulator–target gene pairs (with population association *p*<0.05) interact. We found 75 of our gene pairs in their data (corresponding to 63 unique regulator–target gene pairs; some pairs were found more than once; see Table 4 and Table S7). The chance of finding two genes as regulatory pairs in our mapping study and as interacting partners in the Hi-C experiment by a different group randomly is very small; thus we believe that these are likely true interactions. The Hi-C results further confirm findings from our genetic studies. These data suggest that the regulators and their target genes may be co-transcribed in “transcription factories” [40]–[42].

**Table 4:** Examples of regulator–target gene pairs that interact physically based on Hi-C experiments* [39].

Regulator	Target	*t* Value	SNP (QTDT)	*p* Value (QTDT)	Hi-C Coordinate (Regulator)	Hi-C Coordinate (Target)
*ATRN*	*NFIB*	5.7	rs151507	0.01	chr20:3566294	chr9:14180554
*USO1*	*WDR13*	5.64	rs324734	0.004	chr4:76912713	chrX:48351440
*CTNNBIP1*	*DDX58*	5.63	rs935073	0.0009	chr1:9873467	chr9:32472537
*ROBO1*	*ATF6*	5.38	rs1507417	0.02	chr3:79217935	chr1:160093368
*TMEM45A*	*KCNMA1*	5.06	rs6799992	0.01	chr10:101764442	chr10:78646999
*KCNQ5*	*ETV6*	5.04	rs16883476	0.0002	chr6:73892628	chr12:11800513
*KCNQ5*	*ETV6*	5.04	rs16883476	0.0002	chr6:73522933	chr12:11841656
*KCNMA1*	*HMGCS1*	5.04	rs11002137	0.006	chr10:78782737	chr5:43334692
*KCNMA1*	*HMGCS1*	5.04	rs11002137	0.006	chr10:78778388	chr5:43334974
*PRKCE*	*LIG4*	4.99	rs2711295	0.0004	chr2:45893756	chr13:107664041
*PHLPP*	*PFKL*	4.88	rs2053600	0.03	chr18:58718878	chr21:44555603
*ROBO1*	*VRK2*	4.83	rs9838937	0.001	chr3:79422794	chr2:58139091
*ROBO1*	*VRK2*	4.83	rs9838937	0.001	chr3:79020607	chr2:58189813
*SDCCAG8*	*ACBD3*	4.82	rs11800122	0.008	chr1:241614818	chr1:224416160
*SDCCAG8*	*ACBD3*	4.82	rs11800122	0.008	chr1:241640785	chr1:224442930
*SDCCAG8*	*ACBD3*	4.82	rs11800122	0.008	chr1:241732306	chr1:224420181
*DIS3L2*	*GLTSCR2*	4.82	rs3100608	0.01	chr2:232554756	chr19:52951146
*WWOX*	*IMPA2*	4.82	rs11150104	0.002	chr16:76861753	chr18:11973686
*SDCCAG8*	*NDEL1*	4.81	rs10803140	0.009	chr1:241615074	chr17:8316669
*SMYD3*	*COX4NB*	4.75	rs2105158	0.003	chr1:244566879	chr16:84369002

*This table shows only the regulator-target pairs with the most significant linkage evidence. The complete list is given in Table S7.

Since the Hi-C libraries were not sequenced exhaustively [39], some of our pairs may not be included in their results even though they interact physically. Nevertheless, the results provide additional information for some of the regulatory relationships and show that similar approaches can be used to extend the analysis.

### Characteristics of the *Trans*-Regulators

The resolution of our mapping study allowed us to identify the polymorphic regulators of nearly 1,000 human genes. Instead of just confirming these results computationally, we used molecular approaches, which provide an independent method for assessing the findings. Even though we picked regulatory relationships that had modest statistical support (*p* = 10^−5^ to 10^−2^) from our mapping study, over 70% of the regulatory pairs are validated molecularly. Thus, we are reasonably confident that many of the 1,036 gene pairs have true regulatory relationships, so we went on to characterize them.

First, many of the *trans*-regulators were not known to influence gene expression. Among the 742 regulators, 112 (15%) are known transcription factors and 140 (19%) play a role in signaling pathways. The remaining genes have a variety of functions including metabolism (for example, *MAN2A1*, *PDHA2*) and protein transport or modification in the endoplasmic reticulum (for example, *LMAN1*, *SEC31A*).

Second, the target genes and their regulators often belong to the same functional pathways. For example, midasin (*MDN1*), which plays a role in protein processing [43], regulates the expression of dynactin 1 (*DCTN1*) and signal sequence receptor, delta (*SSR4*). Both of these target genes also participate in protein transport and processing in the endoplasmic reticulum [44],[45]. To test formally whether regulators and their target genes belong to the same functional groups more often than by chance, we annotated the regulators and target genes using Gene Ontology [46]. We found significantly (*p* = 0.008) more regulator–target gene pairs with the identical ontology annotation than random pairs of genes. The criterion we used is quite stringent since we required members of a gene pair to have the identical ontology grouping; it excludes regulator–target gene pairs that are in the same pathway but do not have the identical ontology. However, despite the stringent criterion, a significant result was obtained. Recent results from yeast studies also showed that regulators and their target genes share common gene ontology annotations [47].

Third, many of the *trans*-regulators have more than one target gene. We found 161 (22%) of the 742 *trans*-regulators influence the expression levels of two or more genes. Table 5 shows the 11 regulators that influence six or more target genes. Three of these regulators are known to play a role in transcription regulation through chromatin modification (*AEBP2*) or as transcription factors (*BCL2*, *ZCCHC2*). In addition, three of the regulators (*PHLPP*, *RAMP1*, *WDR7*) affect gene expression through signal transduction pathways. The remaining five regulators are not known to be gene expression regulators, including *TTC5*, which has no known function.

**Table 5:** *Trans*-regulators with six or more target genes.

Regulator	Target Genes
*AEBP2*	*CAPNS1*, *CGRRF1*, *HIVEP1*, *LOC642732*, *MED4*, *PARVB*
*BCL2*	*AES*, *CPNE1*, *PDAP1*, *PMVK*, *TAGLN2*, *ZFPL1*
*KIAA1468*	*CD44*, *F11R*, *HNRPDL*, *MLLT10*, *PRKCSH*, *PSMD12*, *TDG*, *YTHDF2*, *ZWINT*
*KMO*	*ABCF2*, *EIF1*, *HEXB*, *TNFRSF14*, *ZMYM2*, *ZNF330*
*MDN1*	*AES*, *DCTN1*, *EMP3*, *MRPL23*, *PDHX*, *SSR4*
*PHLPP*	*ANXA6*, *CSTF1*, *NRBP1*, *PFKL*, *RAB11B*, *SLC37A4*, *STK19*, *VAV2*
*RAMP1*	*GAPDH*, *LOC645899*, *PLK1*, *RPL23A*, *RPL32*, *RPL41*, *RPS13*, *RPS15*, *RPS17*, *RPS18*, *RPS23*, *RPS24*, *RPS4X*
*TTC5*	*C11orf10*, *GLUD2*, *HSP90AA1*, *RPS3A*, *USP3*, *USPL1*
*USP40*	*ACTG1*, *RPL22*, *RPLP1*, *RPS10*, *RPS3A*, *RS3A*
*WDR7*	*APPBP1*, *ATP5G2*, *BCAS2*, *LOC642732*, *PIK3CB*, *RCC1*, *RHOC*, *SPAG7*, *SYNCRIP*
*ZCCHC2*	*BTBD2*, *CD37*, *CNOT3*, *DDX10*, *EML2*, *GNAI2*, *GNB2*, *PPP2R1A*

### Gene Network

**Figure 4:**
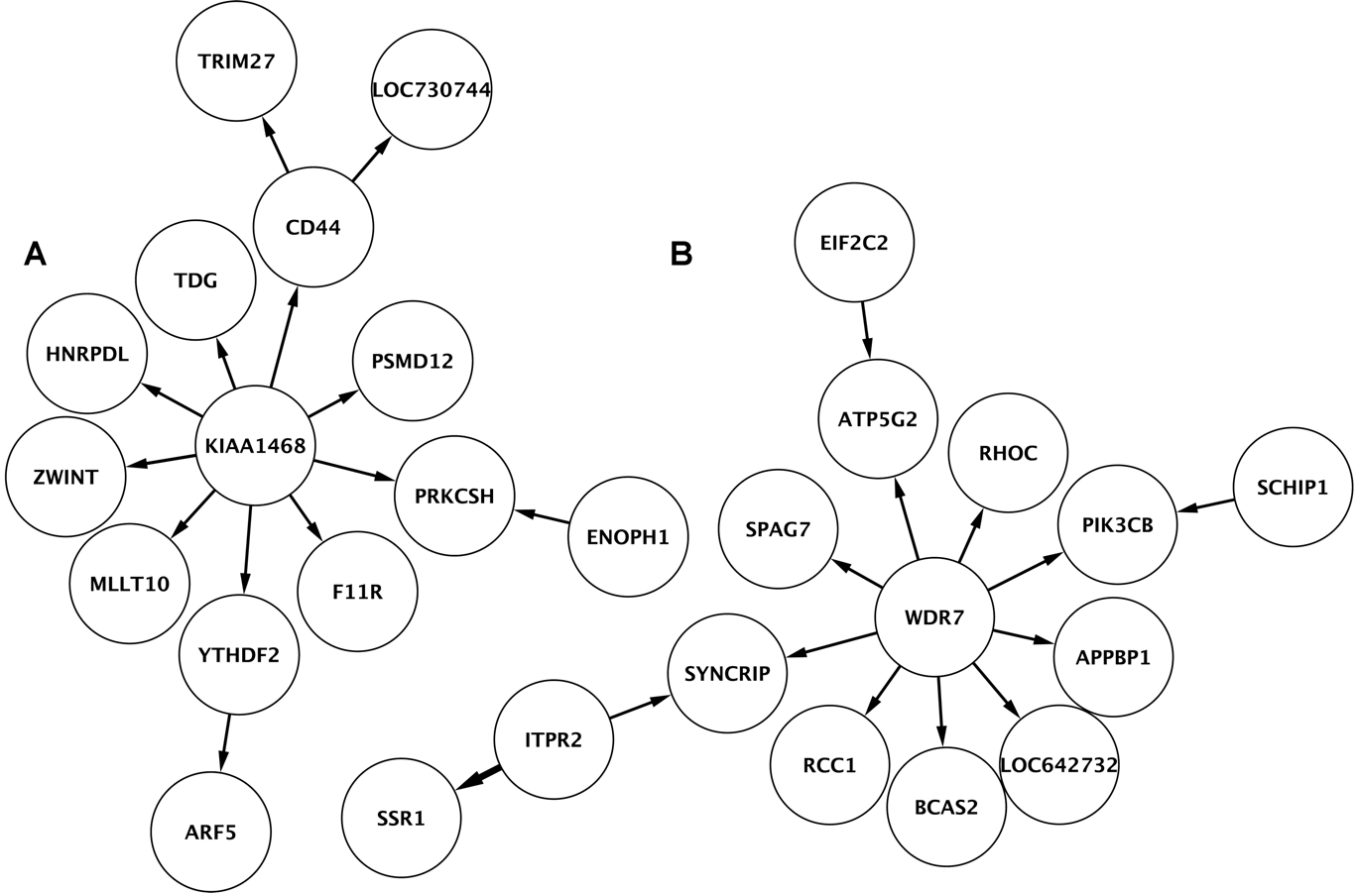
Directed subnetworks. Regulators are connected to their target genes based on results (*p*<0.05) from QTDT analyses. Directions of the arrows go from regulators to their target genes. The two examples correspond to genes connected to KIAA1468, a gene with no known function (a), and *WDR7*, a gene associated with age of onset of multiple sclerosis (b) [51].

The regulators with multiple target genes prompted us to examine interactions beyond the relationship between a gene and its regulator. To do so, we used our mapping results to construct directed gene networks. We connected regulators and their target genes using results from the QTDT analysis. The resulting network consists of 1,036 connections among 742 regulators and 917 target genes. As in many biological networks, the network connections follow a scale-free distribution (scale-free criterion = 0.98) [48]. On average, genes have 1.3 connections, but some genes have more connections such as those that regulate the expressions of several target genes. Figure 4 shows subnetworks for KIAA1468 and *WDR7*, which illustrate that some regulators have multiple target genes and some genes are regulated by more than one regulator. Unlike many gene networks, the nodes in our networks are connected by directed edges based on genetic data. DNA variants in the highly connected genes such as KIAA1468 and *WDR7* influence the expression of many genes that are directly and indirectly connected to them. The *WDR7* subnetwork shows the connections between *ITPR2* and *SSR1*, as well as several other genes, including *SYNCRIP* [49] and *RHOC* [50], that play a role in the endoplasmic reticulum; thus polymorphisms in *WDR7* likely affect protein processing and secretion, the primary functions of the endoplasmic reticulum. Prior to this analysis, the function of *WDR7* was unknown except that it has been found to influence the age of onset of multiple sclerosis [51] in genome-wide association studies. Results from our analyses offer *WDR7* as a mechanistic link between multiple sclerosis and functions of the endoplasmic reticulum. The efficiencies of the endoplasmic reticulum can influence susceptibility to multiple sclerosis in different ways. First, studies have shown that the endoplasmic reticulum plays a key role in immunity, for example in ensuring the maturation of B-cells to immunoglobulin secreting plasma cells [52]. In addition, during myelination, cells such as oligodendrocytes rely on the endoplasmic reticulum to produce a large amount of plasma membrane [53]. Thus by altering the efficiencies of endoplasmic reticulum, variants in *WDR7* can influence individual susceptibility to multiple sclerosis through the autoimmune and/or myelination pathways. Besides *WDR7*, other regulators in our network have also been identified as disease susceptibility genes (see examples in Figure S2).

## Discussion

The main focus of this study is to assess and determine the polymorphic regulation of human gene expression. We used linkage analyses to locate the polymorphic regulatory regions for 1,681 human genes. About 6% to 24% of these regulatory regions were close (proximal) to the target genes, and the remaining regions were further away (distal) from the target genes and mostly on other chromosomes. In follow-up association studies and sequence-based DAE analyses, at least 60% of phenotypes with proximal linkage peaks were found to be *cis*-regulated; this result is similar to findings in yeast [2],[11]. The remaining phenotypes with proximal linkages are likely regulated by *trans*-regulators that are close to their target genes. For 917 genes with distal linkage peaks, we narrowed the regulatory regions and identified the *trans*-acting polymorphic regulators. For some genes, we identified more than one *trans*-regulator; thus, the results include a total of 1,036 regulator–target gene pairs. Previous genetics of human gene expressions studies uncovered only the regulatory regions; here, we improved the resolution significantly by finding the individual regulators.

The results allowed us to explore previously unknown aspects of gene regulation. We found that many genes besides transcription factors can influence the expression of other genes. Similar results were found in yeast [2],[54]. Only 34% of the polymorphic *trans*-regulators that we identified are transcription or signaling factors. Many of the regulators are found in the same functional pathways as their target genes. By eliminating the recruitment of regulators from other pathways, cells can alter gene expression quickly when a cellular process requires a gene to be turned on or off. We do not know yet how polymorphisms in these genes influence expression in *trans*. One possibility is that the sequence variants in or near the regulators affect their own message and protein levels (*cis*-regulation) and lead to differential expression levels of the target genes that they regulate (*trans*-regulation). Based on our RNA-Seq data, ∼20% of the *trans*-regulators show such DAE or *cis*-regulation. Alternately, the sequence variants in the regulators can affect their structures, stabilities [55]–[57], and functions by changing modifications such as phosphorylation status [58]; these in turn can affect the expression of their target genes. We also do not know whether the regulatory relationships are direct or indirect. Since regulatory relationships are highly complex and most genes are regulated by multiple genes in different feedback mechanisms, we expect most regulatory relationships are indirect. The Hi-C data show that some of the regulator–target gene pairs interact physically at the DNA level; the results imply that they may be co-transcribed perhaps in “transcription factories” [40],[41] where others have found *trans* interactions among regulators and their target genes [42].

Although the regulatory mechanisms remain unknown, we found that regulatory relationships are shared among cell types. For a number of genes, the *trans*-regulatory relationships that we identified in immortalized B-cells are also found in primary fibroblasts. Others have found that *cis*-regulation of some genes is shared across cell types [4],[9],[59],[60]; here, we provide evidence that *trans*-regulation can also be shared across different cells. This is important since many cell types in humans are not easily accessible. These results suggest that it may be possible to use more readily available cells for analysis and apply the results across cell types.

Our results have implications beyond regulation of gene expression. It provides insight into disease mechanisms. We already discussed the role of *WDR7* as regulator of genes in the endoplasmic reticulum and the implication of this finding for multiple sclerosis. There are additional examples: for instance, we identified inositol 1,4,5-triphosphate receptor, type 2 (*ITPR2*) as a regulator of signal sequence receptor, alpha (*SSR1*, also known as *TRAPA*). Both genes function in the endoplasmic reticulum [45],[61] and are susceptibility genes for amyotrophic lateral sclerosis (ALS) [62],[63], but the connection between them was previously unknown. By showing the regulatory relationship between these two endoplasmic reticulum genes, we implicate inefficient endoplasmic reticulum function in the development of ALS. The role of the endoplasmic reticulum in ALS is further supported by another regulator–target gene pair, *ALS2* and its target gene, *SEC22A*. *ALS2* is the mutated gene in juvenile ALS [64]. Despite several knockouts of *Als2* in mice, its role in ALS has not been identified. Here, we found that it regulates expression of *SEC22A* (linkage *t* = 5.6, QTDT *p* = 0.004 rs3219171), which mediates endoplasmic reticulum to Golgi transport. These findings have therapeutic implications; a recent study suggested that survival of ALS mice can be extended by blocking endoplasmic reticulum stress induced cell death [65].

Unraveling the control of gene expression of human cells is critical for understanding normal cellular processes and disease mechanisms. It is difficult to identify *trans*-acting regulators. They are not restricted to regulatory genes such as transcription factors. The hundreds of regulators identified in our study do not share protein domains nor belong to particular protein families. Thus, the search cannot be guided by known regulatory functions or protein domains alone. We show that GOGE study along with RNA-Seq and molecular analyses allow the identification of *cis*- and *trans*-acting regulators of human gene expression. This approach makes it possible to determine how individual genes are regulated and to discover regulatory pathways that maintain cellular functions in human cells.

## Materials and Methods

### CEPH Samples, Genotypes, and Expression Phenotypes

The data were from members of 45 three-generations CEPH families (CEPH 1328, 1330, 1331, 1332, 1333, 1334, 1340, 1341, 1344, 1345, 1346, 1347, 1349, 1350, 1353, 1354, 1356, 1357, 1358, 1362, 1375, 1400, 1408, 1413, 1416, 1418, 1420, 1421, 1423, 1424, 1444, 1447, 1451, 1454, 1456, 1458, 1459, 1463, 1477, 1582, 13281, 13291, 13292, 13293, 13294). Low-density genotypes for 4,600 autosomal SNP markers were obtained using the Illumina Linkage Panel (v3). We used PedStats [66] to check for mendelian inconsistencies. This resulted in the removal of 297 genotypes at 209 distinct SNP markers. High-density genotypes for some of the grandparents and parents were obtained from the International HapMap Project (HapMap 22), and for those families who are not part of the HapMap project, the parents and one randomly selected child in each family were genotyped using the Human SNP Array 5.0 (Affymetrix), which assays for ∼500,000 SNP loci throughout the human genome. Then, high density genotypes for family-based association (QTDT) on all subjects were obtained by inference using the low-density genotypes and high-density genotypes on selected individuals [67].

For expression analysis, immortalized B cells were grown at a density of 5×10^5^ cells/mL in RPMI 1640 with 15% fetal bovine serum, 2 mM L-glutamine, and 100 U/mL penicillin-streptomycin. RNA was extracted from the cells and hybridized onto Human Focus Arrays (Affymetrix; ∼8,500 RefSeq Genes on each array). Samples were grown and processed in random order to minimize batch effects. Samples of sibs were processed together only by chance. Expression intensity was scaled to 500 using the global scaling method implemented in the Expression Console software from Affymetrix and transformed by log_2_.

The RNA samples for 94 CEPH grandparents (from the 45 families) were hybridized onto duplicate arrays. This allows us to calculate “variance ratio” as a measurement of variability in expression levels among individuals relative to the measurement noise. For each expressed gene (called “present” by Affymetrix Expression Console in 80% or more grandparents), we calculated this measure as the ratio of the variance in mean expression levels among individuals to the mean of the variance of the replicates within individuals: (variance of *M_i_*)/(mean of *s_i_^2^*). There are 4,793 genes with a variance ratio >1. We focused on these genes in our analyses.

### Analysis of Linkage and Association

Multipoint genome-wide linkage analysis was done by SIBPAL in S.A.G.E. (http://darwin.cwru.edu/) [68]. We used the “W4” option [69] for weighting pairwise phenotypic differences between siblings. SIBPAL determines evidence for linkage at each SNP from regression of the phenotype difference between siblings on the estimated proportion of marker alleles shared identical-by-descent between siblings; the result is reported as a *t* value with corresponding significance. Point-wise significance was converted to genome-wide significance for multiple testing of markers by use of the expression of Lander and Kruglyak (as implemented at http://www.imbs-luebeck.de/8859-15/software/silclod.html) [19]. In SIBPAL linkage analysis, for each family phenotypic data of the children were used, and those for the grandparents and parents were not used.

Family-based association analysis with SNPs near and within the target genes or candidate regulators was carried out using QTDT [21],[70]. We tested about nine genes (median) per (*trans*) linkage peak. Within a gene, the SNP markers are often in strong linkage disequilibrium and thus are not independent tests. We report nominal *p* values for the QTDT results. In the linkage analysis, we used only data from children in the CEPH families; however, in the QTDT analysis, we used data from all members of the CEPH families. For the QTDT, we used the orthogonal (ao) model [21] and variance component options (wega).

We carried out population association analysis to follow-up results of QTDT. For these studies, expression phenotypes from 86 unrelated parents in the 45 CEPH families, as dependent variables, were regressed on SNP genotypes (coded 0, 1, 2). Conventional analysis of linear regression was carried out; we tested SNPs within a gene that showed significant QTDT for each phenotype. To minimize multiple testing, for each significant *trans*-linkage peak, we tested only the gene where the most significant QTDT result was found; SNPs within these genes are mostly highly correlated so we did not consider them as independent tests. We reported the nominal *p* values for these tests.

### RNA-Seq

mRNA-Seq was performed as recommended by the manufacturer (Illumina). Briefly, immortalized B-cells from 41 unrelated CEPH grandparents (part of the International HapMap Project and the 45 families in this study) were grown and processed for RNA-Seq; hence these are biological replicates of those used in our microarray-based analysis. Poly(A) mRNA was extracted using Dynal oligo(dT) beads, fragmented, and first strand cDNA generated using random hexamers. Following second strand cDNA synthesis, end repair, and addition of a single A base, Illumina adaptors were ligated onto the samples. Then, ∼200 bp fractions of the cDNA samples were isolated from agarose gels and PCR amplified. The qualities of the PCR amplicons were checked using the Agilent Bioanalyzer. The samples were then sequenced using the Illumina Genome Analyzer. We obtained an average of 41 million 50 bp reads per sample (median = 40 million).

For alignment of the short reads sequences to the human reference sequence (hg18) and identification of SNPs, we used the program MAQ (version 0.6.8) [25]. To minimize sequence errors, we used the first 40 of the 50 nucleotides in each sequence read for our analysis. For the alignment, we used the default settings of MAQ: allowing up to two mismatches per read. From the aligned reads with map quality scores of 30 or higher, we identified SNPs. For this analysis, we used only known SNPs in dbSNP Build 129. For a sample to be heterozygous at a SNP for our DAE analysis, we required that each allele be represented in at least 5% of the total reads covering that locus. To determine the expression level of a gene, we calculated RPKM [26]. Among our data, ∼700 genes with average RKPM >1 were “called” absent on microarrays. If we had relied on microarray to identify “expressed” genes, these genes and the genes that were not represented on the microarrays would have been excluded in our analyses.

To check the accuracy of our RNA-Seq results, we compared the expression levels with those from our microarray and genotypes from our sequencing data with those obtained by HapMap Consortium. For each gene, we calculated correlation coefficient of the expression levels between the two platforms across the 41 samples. The average correlation coefficient was 0.76 (median = 0.76; range = 0.73 to 0.80). For each sample, we also identified the homozygous genotypes (AA, CC, GG, TT; ∼25,000 genotypes per sample) using the HapMap database and compared them to genotypes in our sequencing results. The comparisons showed a high degree of agreement. Across the 41 samples, the average concordance rate is 98.6% (median = 98.7%).

### Network Analysis

The gene regulatory network was constructed based on pairwise regulatory relationship identified through linkage (*t*>4) and QTDT analyses (*p*<0.05). Connections (edges) were placed between genes that were implicated in a regulator-target interaction. Properties of the resulting gene regulatory network were analyzed in MATLAB (MathWorks) by representing regulatory relationships as an asymmetric adjacency matrix. The number of incoming and outgoing connections per gene was determined by summing the columns and rows of the adjacency matrix. A MATLAB function for determining the scale-free topology criteria was implemented as previously described [48]. Code will be provided upon request. Figures of the resulting networks were drawn using Cytoscape 2.6.0 [71]. To identify genes that have been implicated as human disease susceptibility genes, we queried the Catalogue of Genome-Wide Association Studies (http://www.genome.gov/26525384) [72].

### Functional Pathway Analysis

To determine whether a regulator and its target belong to the same functional groups, we examined Gene Ontology Biological Process terms [46] for the regulator and the target genes. We counted the number of regulator-target pairs with identical Gene Ontology Biological Process annotations; these were the “observed counts.” We then examined 1,000 randomly chosen gene pairs (from expressed genes in our B-cells) and counted the number of gene pairs that shared Gene Ontology Biological Process annotations. We repeated this 5 times and took the average; these were the “expected counts.” We compared the observed to the expected counts by a chi-square test.

### Knockdown of Candidate Regulators

Immortalized B cells of four to six individuals and primary fibroblasts (foreskin) from two healthy newborns were used. The cells were transfected with Accell siRNAs (Thermo Scientific) against candidate regulators or non-target control according to the manufacturer's instructions. For each regulator, we used a pool of siRNAs that target the regulators in order to minimize off-target effects [36]. To compare the knockdown by pools of siRNA and single siRNAs against a gene, we silenced GPHN using a pool of siRNA and 2 siRNAs against different parts of the gene; similar results were obtained in the three experiments (see Table S3B).

For each transfection, immortalized B cells were seeded at a concentration of 4.5×10^5^ cells per 750 ul on the day of transfection. 7.5 ul of 100 uM Accell siRNA was mixed with the seeded culture. Each transfection mix was then plated in a 96-well tissue culture plate in 150 ul aliquots. Similarly, 4.5×10^5^ cells per 750 ul of primary fibroblasts were plated in 12-well plates in growth media the day before the transfection. On the day of transfection, the growth media were removed and replaced with 7.5 ul 100 um Accell siRNA (against genes of interest or scrambled sequence as control) and 750 ul of Accell media.

The transfected cells with siRNAs were incubated at 37°C for 96 h. We then replaced the Accell media with regular growth media (RPMI 1640 with 15% fetal bovine serum, 2 mM L-glutamine, and 100 U/mL penicillin-streptomycin) and let the cells recover for 24 h. RNA was extracted using Qiagen RNeasy kits. Effects of siRNA on gene expression were analyzed by quantitative PCR (Applied Biosystems). Expression of ACTB was used for normalization and changes in expression were calculated relative to cells transfected with non-target control siRNA. Sequences of PCR primers and siRNAs are presented in Tables S4 and S5.

### Insulin Treatment

For the western analysis: primary fibroblasts were cultured in MEM medium supplemented with 10% fetal bovine serum, 2 mM L-glutamine, and 100 U/mL penicillin-streptomycin. Cells were serum starved for 18 h before treatment with 100 nM insulin for 5 min. Cells were lysed in 1× Lysis buffer (20 mM Tris-HCl pH 7.5, 150 mM NaCl, 1 mM Na_2_EDTA, 1 mM EGTA, 1% Triton ×100) (Cell Signaling) supplemented with 1× Complete protease inhibitors (Roche) and 1× phosphatase inhibitors I and II (Sigma). Cell lysates containing 150 µg of total protein were incubated with 5 µg of α-INSR antibody (#3025, Cell Signaling) or α-IGF1R antibody (#3018, Cell Signaling) at 4°C overnight. Immuno-complex was pulled down using Protein A Sepharose (GE Healthcare). Input and immunoprecipitation samples were analyzed by Western Blot using α-phosphotyrosine (1∶1000) (4G10 Platinum, Millipore) or the above α-INSR (1∶1000) and α-IGF1R (1∶1000) antibodies.

For the gene expression analysis, primary fibroblasts (from foreskin) of four individuals were cultured as above. Cells were serum starved overnight (18 h) before being treated with 100 nM insulin (Sigma) for 0, 1, 2, 6, or 12 h. RNA was extracted and gene expression was analyzed by quantitative PCR (Applied Biosystems). Sequences of PCR primers are presented in Table S6.

### Comparison to Hi-C Data

Hi-C data from Dekker and colleagues [39] were obtained from NCBI GEO (GSE189199); we used the data in their alignment summaries. We compared their list of interacting pairs to our regulator–target gene pairs. A match is called when one of their interacting pair coordinates was found within a regulator or 5 kb up- or downstream and the matching member of that pair is found within or 5 kb up- or downstream of the corresponding target gene. Seventy-five such pairs were found.

The experimental steps in this study are summarized by a flowchart (Figure S3).

#### Accession Numbers

The data have been deposited to NCBI GEO under the accession numbers GSE16778 and GSE16921 for the microarray and RNA-Seq data, respectively.

## Supporting Information

Figure S1 **Differential allelic expression by RNA-Seq.** Allelic expression ratio of 273 exonic SNPs in 43 genes. Data for each heterozygous individual is represented as a color dot. SNPs are ordered left to right by mean expression ratio, a/(a+b).
(5.83 MB TIF)

Figure S2 **Examples of subnetworks that include genes that were implicated as disease susceptibility in genome-wide association (GWA) studies (** http://www.genome.gov/gwastudies/ **).**
(1.65 MB PNG)

Figure S3 **Flowchart showing experimental steps.**
(0.08 MB PDF)

Table S1 **Linkage, QTDT, and association results for** ***cis*** **-regulated genes.**
(0.07 MB PDF)

Table S2 **Regulators for 200** ***trans*** **-regulated expression phenotypes.**
(0.06 MB PDF)

Table S3 **Additional results of knockdown of** ***trans*** **-regulators.** (A) Knockdown of *trans*-regulators in fibroblasts. (B) GPHN knockdown using a pool of 4 siRNAs compared to individual siRNAs.
(0.10 MB PDF)

Table S4 **Primer sequences for qRT-PCR (gene knockdown experiment).**
(0.04 MB PDF)

Table S5 **siRNA sequences (gene knockdown experiment).**
(0.05 MB PDF)

Table S6 **Primer sequences for qRT-PCR (insulin treatment).**
(0.03 MB DOC)

Table S7 **Regulator–target gene pairs that are found to interact physically by Hi-C.**
(0.05 MB PDF)

## Abbreviations

3C: chromosome conformation capture
ALS: amyotrophic lateral sclerosis
CEPH: Centre d'Etude du Polymorphisme Humain
DAE: differential allelic expression
FDR: false discovery rate
Gb: gigabase
GOGE: genetics of gene expression
LOD: logarithm of odds
QTDT: quantitative transmission disequilibrium test
RNASeq: RNA-sequencing, siRNA, short interfering RNA
SNP: single nucleotide polymorphism
