# Supplementary figures and images for: Polymorphic Cis- and Trans-Regulation of Human Gene Expression
Source: PLoS Biol. 2010 Sep 14;8(9):e1000480. doi: 10.1371/journal.pbio.1000480 (PMC2939022; doi:10.1371/journal.pbio.1000480)

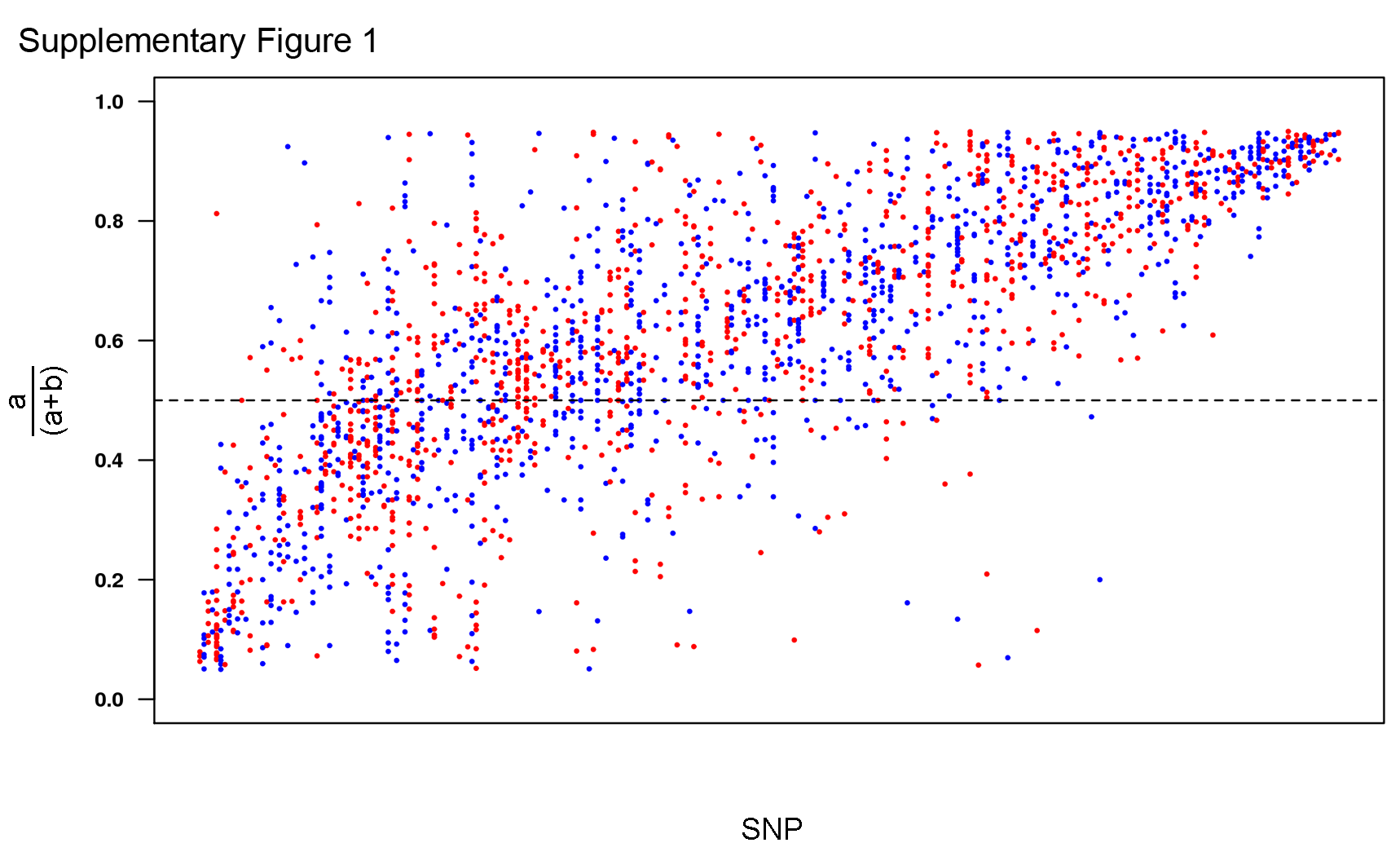

Supplement: Figure S1 — Differential allelic expression by RNA-Seq. Allelic expression ratio of 273 exonic SNPs in 43 genes. Data for each heterozygous individual is represented as a color dot. SNPs are ordered left to right by mean expression ratio, a/(a+b). (5.83 MB TIF) [file pbio.1000480.s001.tif]

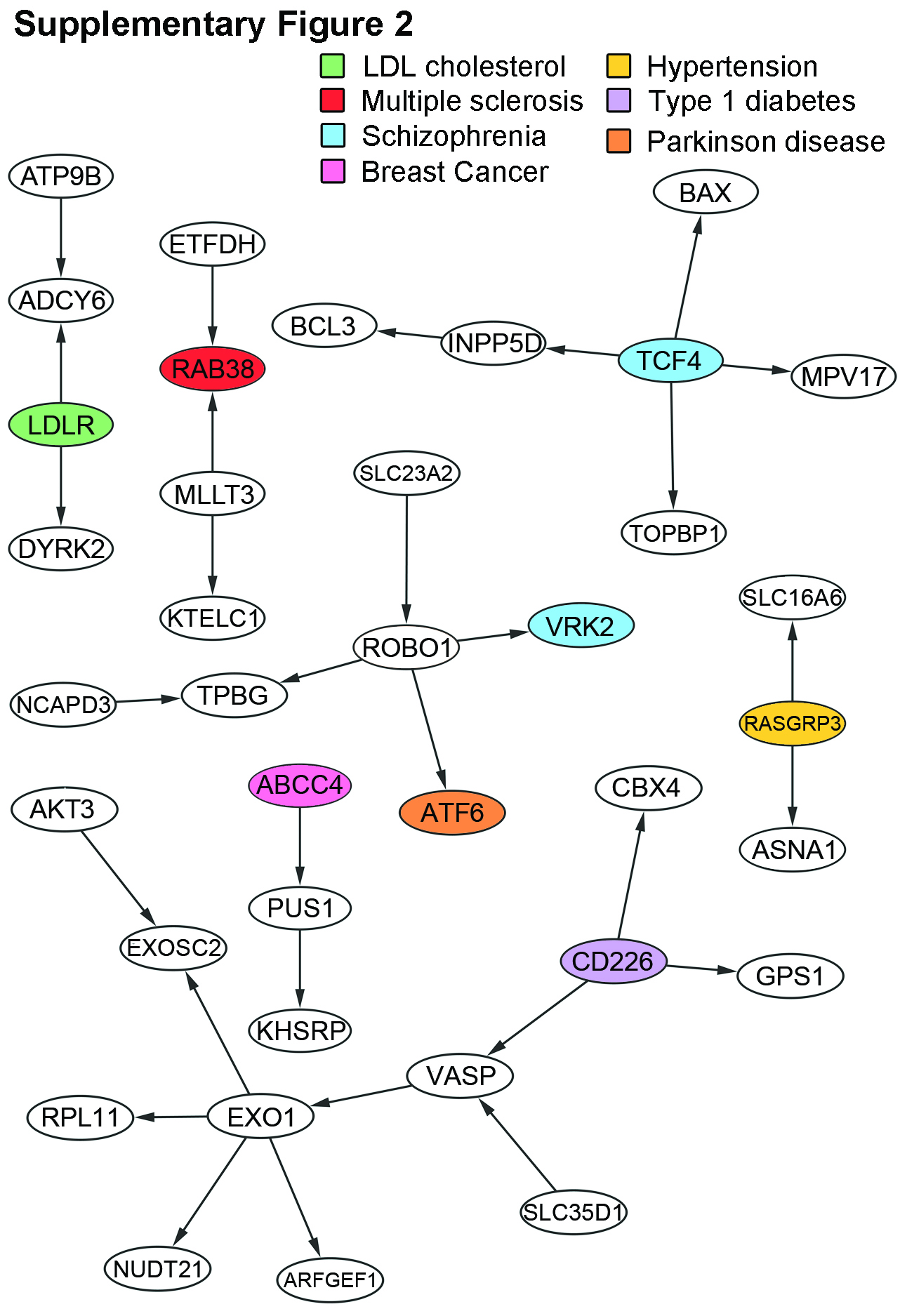

Supplement: Figure S2 — Examples of subnetworks that include genes that were implicated as disease susceptibility in genome-wide association (GWA) studies ( http://www.genome.gov/gwastudies/ ). (1.65 MB PNG) [file pbio.1000480.s002.jpg]

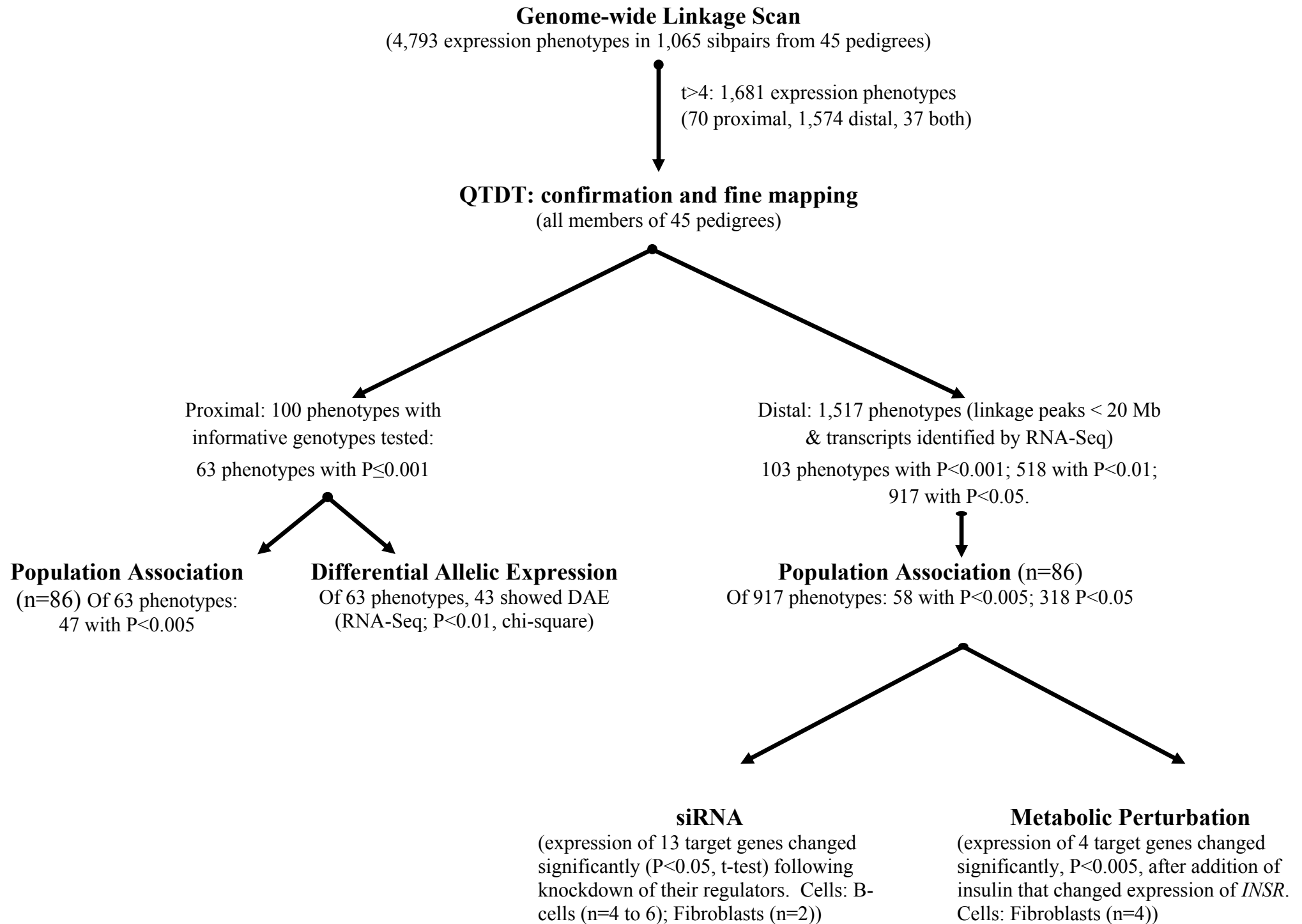

Supplement: Figure S3 — Flowchart showing experimental steps. (0.08 MB PDF) [file pbio.1000480.s003.pdf]
