# Supplementary material for: Polymorphic Cis- and Trans-Regulation of Human Gene Expression
Source: PLoS Biol. 2010 Sep 14;8(9):e1000480. doi: 10.1371/journal.pbio.1000480 (PMC2939022; doi:10.1371/journal.pbio.1000480)
Supplement: Table S1 — Linkage, QTDT, and association results for cis -regulated genes. (0.07 MB PDF) [file pbio.1000480.s004.pdf]

Supplementary Table 1. Linkage, QTDT, association results for cis-regulated genes					
Gene Name	t_linkage	qtdt_rs_id	qtdt_p_value	association_rs_id	association_p
AMFR	9.5	rs2432540	2.00E-06	rs2432540	2.15E-10
AP3S2	5.7	rs3759831	5.00E-07	rs3759831	2.70E-05
ARF6	4.2	rs3100876	2.00E-05	rs3126183	3.64E-03
B4GALT1	4.8	rs10813950	8.00E-06	rs10758189	7.26E-03
BFSP2	5.2	rs6806169	6.00E-04	rs6806169	>0.05
BTN3A2	5.5	rs9379851	1.00E-13	rs9379851	4.51E-10
BTN3A2	5.5	rs9358945	1.00E-04	rs3799380	2.23E-04
CCT8	4.4	rs2832159	2.00E-06	rs2832159	3.00E-03
CHI3L2	8.8	rs11102223	6.00E-11	rs10494129	>0.05
CPNE1	7.2	rs2425071	4.00E-17	rs2425071	1.69E-09
CRYZ	4.8	rs1475396	6.00E-06	rs1475396	2.48E-05
CSTB	6.0	rs2329573	3.00E-08	rs2329573	3.75E-10
CTA-126B4.3	4.6	rs5751297	4.00E-05	rs5751297	1.81E-04
CTBP1	6.6	rs1250105	1.00E-05	rs1250105	5.03E-03
CTSH	4.6	rs3825932	4.00E-08	rs3825932	5.80E-07
DDX42	5.7	rs2665795	1.00E-07	rs2665797	4.11E-04
DFNA5	5.2	rs754553	3.00E-06	rs754553	7.62E-03
DNAJC15	6.3	rs9533377	2.00E-09	rs9533377	1.16E-05
EPS15	5.4	rs6588405	2.00E-06	rs6588405	4.80E-03
GNA12	4.2	rs1182151	2.00E-04	rs1182151	5.41E-03
GPR109B	4.4	rs1798192	1.00E-06	rs1798192	8.51E-04
GSR	4.0	rs3779647	6.00E-04	rs3779647	3.10E-02
GSTM1	8.4	rs7544426	1.00E-03	rs7544426	>0.05
GSTM2	8.9	rs7544426	3.00E-04	rs7544426	>0.05
GSTT1	8.4	rs738809	4.00E-08	rs738809	4.87E-10
HBS1L	7.1	rs4896118	2.00E-18	rs4289677	1.06E-17
HERC1	4.3	rs925251	3.00E-06	rs925251	2.11E-02
HLA-DPB1	10.5	rs9277542	3.00E-35	rs2068204	>0.05
HLA-DQB1	14.4	rs9272346	2.00E-50	rs9272219	9.94E-04
HSD17B12	8.1	rs939015	3.00E-15	rs10838186	1.20E-08
IL16	4.1	rs4076201	1.00E-03	rs4076201	>0.05
IPP	5.2	rs4660313	1.00E-09	rs4660313	2.13E-09
IRF5	9.3	rs2172876	3.00E-08	rs2172876	>0.05
LOC388796	9.1	rs788350	3.00E-36	rs788350	5.18E-19
LRAP	14.6	rs3909451	2.00E-42	rs3909451	1.20E-26
LY86	5.4	rs1330803	7.00E-05	rs1330803	2.07E-05
MTHFD2	4.2	rs702462	2.00E-05	rs702462	>0.05
MTRR	8.2	rs162030	1.00E-04	rs162030	1.07E-03
MXRA7	7.7	rs2159358	1.00E-05	rs2159358	2.23E-03
NAGK	5.4	rs7558220	7.00E-04	rs7558220	1.36E-04
NSMAF	5.2	rs12680996	3.00E-04	rs12680996	2.28E-03
NT5C2	7.0	rs10883826	4.00E-15	rs10883826	3.20E-05
PACSIN2	4.5	rs1040427	2.00E-09	rs7511534	1.91E-04
PAPSS1	4.5	rs2158179	7.00E-04	rs2158179	2.67E-02
PARP4	6.2	rs7995492	4.00E-08	rs7995492	2.06E-04
PEX6	8.9	rs6941212	3.00E-14	rs6941212	1.54E-11
PHYH	5.5	rs17601017	5.00E-09	rs17601017	1.55E-04
POLR1D	5.1	rs10492487	2.00E-07	rs10492487	3.51E-04
PPAT	10.3	rs9683679	3.00E-11	rs9683679	1.52E-09
PSPH	11.6	rs11238381	3.00E-16	rs11238381	8.97E-10
RAB31	4.2	rs1378528	3.00E-07	rs1378528	3.78E-03
S100A13	5.0	rs7345	3.00E-11	rs7345	8.45E-05
SERPINB10	5.5	rs1015416	4.00E-14	rs1015416	2.06E-09
SURF1	7.0	rs1179037	3.00E-06	rs1179037	3.97E-02
TAP2	7.2	rs2071474	7.00E-13	rs2071474	>0.05
TCEA1	7.0	rs11997954	2.00E-07	rs11997954	2.19E-05
TPP2	5.5	rs4772494	7.00E-09	rs4772494	1.36E-11
TRA2A	4.9	rs4719732	6.00E-04	rs4719732	>0.05
UPF3A	5.3	rs9562187	1.10E-03	rs9285061	>0.05
UROS	4.0	rs11244667	8.00E-06	rs11244667	9.67E-04
VAMP8	5.6	rs2043675	4.00E-11	rs2043675	1.63E-09
YWHAE	4.1	rs902966	1.00E-09	rs902966	7.42E-04
ZNF85	7.5	rs17618127	6.00E-10	rs17618127	6.35E-04
