# Supplementary material for: Polymorphic Cis- and Trans-Regulation of Human Gene Expression
Source: PLoS Biol. 2010 Sep 14;8(9):e1000480. doi: 10.1371/journal.pbio.1000480 (PMC2939022; doi:10.1371/journal.pbio.1000480)
Supplement: Table S2 — Regulators for 200 trans -regulated expression phenotypes. (0.06 MB PDF) [file pbio.1000480.s005.pdf]

Supplementary Table 2. 200 expression phentoypes with the most significant evidence of linkage and association to polymorphic trans-regulators

target gene	target gene (chr)	t (linkage)	Regulator	regulator (chr)	qtdt_rs_id	qtdt_p
AATF	17	5.16	GPATCH1	19	rs10411529	5.00E-04
ADAM19	5	4.11	CTSO	4	rs10027171	7.00E-04
AES	19	4.25	MDN1	6	rs6910112	1.30E-03
AKAP7	6	4.48	ABHD6	3	rs4681830	1.30E-03
ALAS1	3	4.16	AGPAT3	21	rs4818873	1.90E-03
ALDH18A1	10	4.49	CUX1	7	rs10252737	1.40E-03
APP	21	4.26	TSPAN17	5	rs1061799	7.00E-04
APPL1	3	4.55	FAM129A	1	rs861615	8.00E-04
ARFGEF2	20	4.43	SNX29	16	rs13336249	1.20E-03
ARHGAP4	X	4.63	PAF1	19	rs10403504	7.00E-04
ARL4C	2	4.5	RAD51L1	14	rs2525525	1.40E-03
ASB1	2	4.68	PDIA5	3	rs836847	1.40E-03
ATP6AP2	X	4.99	SMYD3	1	rs6674691	1.80E-03
ATXN2	12	4.86	NFATC2	20	rs4811172	2.00E-04
ATXN2L	16	4.21	ENOPH1	4	rs1980187	2.00E-05
BCL2A1	15	4.62	PRDM2	1	rs1015370	6.00E-04
BIN3	8	4.55	ESCO2	8	rs4732754	1.70E-03
BRD4	19	4.25	CDC42EP3	2	rs4670748	1.20E-03
C8orf55	8	4.21	SEC22A	3	rs12493729	9.00E-04
CDK2AP2	11	4.61	PPM1B	2	rs7595470	8.00E-04
CHI3L2	1	4.65	C10orf11	10	rs16932960	1.10E-03
CHST10	2	4.49	GCN1L1	12	rs7134706	1.90E-03
CINP	14	5.17	DDRGK1	20	rs6051638	5.00E-04
CLPX	15	4.68	FUT8	14	rs17181388	1.10E-03
COIL	17	5.45	DENND3	8	rs307731	1.80E-03
COPS7B	2	4.76	DERA	12	rs16911499	1.80E-03
CREB3	9	5.73	MIF4GD	17	rs4789161	2.10E-03
CROT	7	4.4	SMARCA2	9	rs7851702	1.50E-03
CUL2	10	4.45	CLDND1	3	rs1729975	1.00E-03
DDX10	11	4.52	ZCCHC2	18	rs17668044	1.10E-03
DDX41	5	4.22	MARK2	11	rs10897458	1.60E-03
DDX58	9	5.63	CTNNBIP1	1	rs935073	9.00E-04
DHRS3	1	4.43	TSEN2	3	rs17036821	9.00E-04
DLEU1	13	4.17	DNAJC24	11	rs17632324	2.20E-03
DLG5	10	5.21	TNIK	3	rs9814699	2.00E-05
DNM2	19	4.4	ZNF407	18	rs7237619	3.00E-04
DUSP14	17	5.97	DNMBP	10	rs11190338	2.00E-03
DYRK2	12	5.82	LDLR	19	rs688	1.90E-03
EDNRA	4	4.95	TRAM2	6	rs3804498	6.00E-04
EGFL6	X	6.33	MCM6	2	rs4988189	3.00E-06
EIF3S9	7	4.48	CAMSAP1L1	1	rs10920017	1.80E-03
EIF5A	17	5.05	PDHA2	4	rs9684694	2.10E-03
ESRRA	11	5.02	SOD2	6	rs8031	7.00E-04
ETAA1	2	4.48	EIF2B5	3	rs4575925	7.00E-04
ETV6	12	5.04	KCNQ5	6	rs16883476	2.00E-04
EVC	4	4.5	C16orf62	16	rs2285663	1.60E-03
EXOC5	14	5.35	C14orf143	14	rs4143999	1.40E-03
FAH	15	4.51	TARBP2	12	rs784567	5.00E-04
FDPS	1	4.12	SLFN11	17	rs7212322	8.00E-04
FEM1B	15	4.35	SLC2A5	1	rs6667506	1.70E-03
FILIP1L	3	4.64	PSIP1	9	rs741930	1.30E-03
FJX1	11	4.17	C20orf94	20	rs589905	2.20E-03
FOXG1B	14	4.09	C10orf11	10	rs2395336	5.00E-04
FOXG1B	14	4.44	NAPB	20	rs2424534	2.00E-05
FPGT	1	4.89	ADIPOR2	12	rs767870	1.80E-03
FRAG1	11	4.31	ALG14	1	rs6698046	8.00E-04
GALNT1	18	4.67	ADIPOR2	12	rs10744552	1.40E-03
GALNT1	18	4.84	RDH14	2	rs7604190	1.80E-03
GAS2	11	4.28	CHMP4B	20	rs4911368	2.00E-03

GCS1	2	4.12	FANCC	9	rs554879	1.40E-03
GMPS	3	4.16	ZNF45	19	rs388685	1.30E-03
GNAI2	3	4.55	ZCCHC2	18	rs17668044	1.50E-03
GNG11	7	4.93	BIRC5	17	rs12947167	1.20E-03
GNPAT	1	4.2	TMCO1	1	rs6426937	1.60E-03
GPR175	3	5.45	TNFRSF11A	18	rs3017359	6.00E-04
GTF2IRD1	7	5.25	TMTC4	13	rs1283198	8.00E-04
HMGCS1	5	4.68	ETV6	12	rs10772498	1.40E-03
HSD17B10	X	4.09	ABHD2	15	rs11858720	4.00E-04
HSP90B1	12	4.95	PEPD	19	rs3786913	3.00E-04
IDH2	15	4.43	ELOVL6	4	rs17041386	1.20E-03
IFNA2	9	4.81	C3orf1	3	rs1967621	1.00E-04
IGFBP4	17	4.51	NCKAP1L	12	rs11170946	4.00E-04
IMPA2	18	4.82	WWOX	16	rs11150104	1.80E-03
IQGAP2	5	4.23	VPS41	7	rs17171473	1.20E-03
IVNS1ABP	1	4.43	CDV3	3	rs4854587	7.00E-04
JTV1	7	4.48	C9orf82	9	rs17760820	1.30E-03
KCND2	7	4.84	XRCC1	19	rs3213266	4.00E-04
KCNMB3	3	4.57	IPO8	12	rs3910561	2.00E-04
KHDRBS3	8	4.72	FAM120B	6	rs910424	4.00E-05
KHSRP	19	4.48	PUS1	12	rs7978708	1.90E-03
LENG4	19	4.81	SNAP23	15	rs9302112	1.80E-03
LIG4	13	4.99	PRKCE	2	rs2711295	4.00E-04
LIN7A	12	4.49	YLPM1	14	rs2241275	2.00E-04
LMO1	11	4.58	ITPK1	14	rs3825684	6.00E-04
LOC730744	4	5.45	CD44	11	rs16927009	7.00E-04
LRRFIP2	3	4.27	TEP1	14	rs11160462	1.10E-03
MAP3K6	1	4.07	TOPORS	9	rs597131	1.50E-03
MED4	13	4.89	AEBP2	12	rs2652013	7.00E-04
MGEA5	10	4.34	MACROD2	20	rs17775664	1.30E-03
MLYCD	16	4.64	DNAJC25-GNG10	9	rs1322251	1.00E-04
MNDA	1	4.41	ATF3	1	rs10494953	7.00E-04
MRPS12	19	4.54	METAP1	4	rs10084896	7.00E-04
NAGA	22	5.2	MEF2B	19	rs7360000	1.60E-03
NAP1L2	X	5.66	ERICH1	8	rs9314603	5.00E-04
NARS	18	4.72	SFRS8	12	rs4964997	1.40E-03
NCKAP1	2	4.77	SLC35B2	6	rs504697	8.00E-04
NCR3	6	4.6	C6orf145	6	rs6930339	1.50E-03
NEDD8	14	4.49	SERBP1	1	rs12566098	1.30E-03
NFATC3	16	4.03	RNPC3	1	rs7529144	9.00E-04
NFYB	12	5.03	UBTD2	5	rs17074475	3.00E-04
NMT2	10	5.01	LRRC8D	1	rs17130931	1.20E-03
NOD2	16	4.86	RBM19	12	rs11609560	1.00E-03
NPAT	11	4.44	RFTN1	3	rs689953	4.00E-04
NRD1	1	4.83	VISA	20	rs16988997	1.60E-03
NT5E	6	4.63	PREP	6	rs9320114	5.00E-04
P2RY5	13	5.21	PSMD8	19	rs2074981	6.00E-04
P4HA1	10	4.21	SFRS18	6	rs13198933	1.60E-03
PAQR4	16	4.08	TTC17	11	rs11605997	1.90E-03
PARP2	14	4.49	CLIP1	12	rs907485	1.40E-03
PARVA	11	4.23	VGLL4	3	rs6807423	2.00E-05
PCNP	3	4.37	SIPA1L3	19	rs3745945	9.00E-04
PCTP	17	4.4	ZFR	5	rs2963980	6.00E-04
PDAP1	7	4.78	BCL2	18	rs10503078	1.20E-03
PDCD10	3	7.36	C4orf41	4	rs4401492	3.00E-04
PDCD10	3	4.72	ZNF429	19	rs2650825	1.00E-04
PDE4B	1	5.89	MBP	18	rs9959822	3.00E-05
PDHA1	X	5.21	ZNF45	19	rs407731	1.30E-03
PDLIM2	8	5.05	DOCK2	5	rs6893165	3.00E-04
PECAM1	17	5.54	PSMD8	19	rs2074981	2.00E-05
PHB2	12	4.2	RUFY3	4	rs6828698	1.80E-03
PILRA	7	4.7	SNRPB	20	rs6049206	5.00E-04
PKN2	1	4.6	NUP50	22	rs9615052	1.50E-03
PLD3	19	4.65	RTTN	18	rs17805883	9.00E-04
PNMA1	14	4.57	TSEN2	3	rs2596827	1.30E-03

POLE2	14	4.31	MLXIP	12	rs10847853	4.00E-04
POLR1C	6	4.82	IFT74	9	rs7035447	1.80E-03
PPIF	10	4.36	CDKAL1	6	rs6915209	2.00E-03
PPM1G	2	4.64	ZFYVE26	14	rs12881478	5.00E-04
PPP1R10	6	4.9	SMYD3	1	rs10754492	1.60E-03
PPP2R5D	6	4.21	FAS	10	rs10509561	3.00E-04
PRKCSH	19	4.82	ENOPH1	4	rs10516674	2.10E-03
PSCD2	19	5.24	GNA15	19	rs2074865	1.30E-03
PSG1	19	4.65	ARL8A	1	rs17488131	1.10E-03
PSMB10	16	4.02	ZNF85	19	rs11085405	9.00E-04
PSMD14	2	4.83	FECH	18	rs8094527	1.10E-03
PSMD4	1	5.5	WWOX	16	rs9936829	1.90E-03
PSPH	7	4.98	IL1R1	2	rs3917328	1.00E-03
PTGIR	19	4.61	DYM	18	rs1943000	4.00E-04
PTPRG	3	4.15	GALNTL4	11	rs9943596	9.00E-04
PYCR1	17	4.3	ARHGAP15	2	rs354694	3.00E-04
RABEP1	17	4.46	ANKS1A	6	rs3800426	1.50E-03
RAF1	3	4.36	PEPD	19	rs17834067	3.00E-04
RASSF1	3	4.43	SIPA1L3	19	rs17249336	2.00E-04
RASSF3	14	5	UBA6	4	rs10010188	1.30E-03
RCN1	11	4.38	RPL7L1	6	rs2894484	1.30E-03
RHOB	2	4.3	ACBD3	1	rs9660924	1.30E-03
RNMT	18	4.82	SLC1A1	9	rs4742003	1.60E-03
RPL22	1	4.56	USP40	2	rs1597940	2.00E-03
RPLP1	15	4.18	USP40	2	rs1597940	6.00E-04
RPP40	6	5.09	DYM	18	rs16950298	1.50E-03
RPS10	16	4.54	USP40	2	rs1597940	5.00E-04
RPS3A	4	5.05	USP40	2	rs1597940	3.00E-04
RPS6KC1	1	4.77	DHRS3	1	rs4846126	1.30E-03
RSU1	10	4.13	FAM53B	10	rs10901802	2.00E-04
SDHD	11	5.31	GALNT7	4	rs10471199	8.00E-04
SEC61B	9	5.18	TBC1D5	3	rs17043638	1.40E-03
Sep9'	17	4.63	CCDC77	12	rs735295	2.20E-03
SF3A3	1	4.62	ZNF407	18	rs17243661	7.00E-04
SFXN3	10	5	C1orf27	1	rs12084264	6.00E-04
SH2B3	12	5.43	CUGBP2	10	rs7896383	1.10E-03
SIAH1	16	4.55	SNRPF	12	rs3751264	3.00E-04
SLC25A32	8	4.87	IPO7	11	rs4910052	3.00E-04
SLC37A4	11	4.7	PHLPP	18	rs2053600	1.30E-03
SMARCD1	12	4.08	SUMF1	3	rs17040638	1.50E-03
SMC3	10	4.73	ZNF100	19	rs12972593	7.00E-04
SNPH	20	4.73	ZCCHC7	9	rs17408510	1.40E-03
SNX10	7	4.45	ZDHHC17	12	rs11115332	4.00E-04
SPHK2	19	5.85	NHEJ1	2	rs17574343	5.00E-04
SPHK2	19	5.81	ATG16L2	11	rs4944804	2.10E-03
SSR1	6	4.58	ITPR2	12	rs12823128	2.00E-05
STAT1	2	4.82	ANK1	8	rs474051	1.50E-03
STS	X	4.94	SLC15A2	3	rs17203299	1.30E-03
STXBP3	1	4.38	DCUN1D2	13	rs3814254	1.00E-04
SUV39H1	X	4.69	SP110	2	rs1427290	2.00E-04
SYNGR2	17	4.75	ZNF155	19	rs415521	7.00E-04
TMEM165	4	4.53	RBM19	12	rs10850231	3.00E-04
TNFRSF11A	18	5.07	ABCC1	16	rs8054156	1.60E-03
TNFRSF11B	8	4.97	ITPKB	1	rs3754387	5.00E-04
TOP3A	17	4.68	USP30	12	rs6606733	1.20E-03
TOR3A	1	4.43	AMMECR1L	2	rs7574303	8.00E-04
TRIM33	1	4.19	ANKH	5	rs16903723	7.00E-04
TRPV2	17	4.09	ZW10	11	rs17614161	1.20E-03
TSG101	11	4.12	FHIT	3	rs1735475	6.00E-04
TYMS	18	4.96	OPTN	10	rs17512962	2.00E-05
USF2	19	4.84	SFMBT2	10	rs7080643	2.00E-04
USP1	1	4.05	UXS1	2	rs17279736	3.00E-05
USPL1	13	5.16	KAT2B	3	rs12488369	1.30E-03
USPL1	13	4.68	COG1	17	rs1026129	9.00E-05
VPS45	1	5.17	C1orf57	1	rs10429825	7.00E-04

VRK2	2	4.83	ROBO1	3	rs9838937	1.20E-03
XRCC4	5	4.36	COMMD10	5	rs10042163	7.00E-04
ZBED5	11	4.72	IFT52	20	rs16988169	3.00E-04
ZNF136	19	5.16	RAP1GDS1	4	rs3775537	1.50E-03
ZNF174	16	4.68	CCDC88C	14	rs747053	1.70E-03
ZNF189	9	4.85	ARHGAP10	4	rs6822971	8.00E-05
ZNF202	11	4.18	DHCR24	1	rs608458	1.70E-03
ZNF217	20	4.33	POLK	5	rs5744724	9.00E-04
ZNF706	8	4.99	TMCC3	12	rs2449663	1.10E-03
ZNHIT4	2	4.32	N4BP2	4	rs9993439	1.50E-03
ZWINT	10	4.7	KIAA1468	18	rs17069686	3.00E-04
