# Supplementary material for: Polymorphic Cis- and Trans-Regulation of Human Gene Expression
Source: PLoS Biol. 2010 Sep 14;8(9):e1000480. doi: 10.1371/journal.pbio.1000480 (PMC2939022; doi:10.1371/journal.pbio.1000480)
Supplement: Table S3 — Additional results of knockdown of trans -regulators. (A) Knockdown of trans-regulators in fibroblasts. (B) GPHN knockdown using a pool of 4 siRNAs compared to individual siRNAs. (0.10 MB PDF) [file pbio.1000480.s006.pdf]

Supplementary Table 3A. Results of knockdown of trans-regulators in fibroblasts

Regulator-Target gene*	Changes in expression levels of		
	Regulator†	Target Gene†	Control (GAPDH) ‡
BLM-NUSAP1	-39.1±8.7	13.8±2.2	0.6±5.1
ITGB4BP-SLC25A11	-44.5±3.6	20.6±5.3	0.8±1.5

Supplementary Table 3B Results showing that knockdown of GPHN was achieved with two oligonucleotides (a and b) that target different portions of GPHN and a pool of 4 oligonucleotides (p) that target different parts of GPHN as in Table 2 in main text. Following GPHN knockdown, significant changes in expression of its target gene, RALB, were observed but the expression of a non-target control gene, GAPDH, did not change significantly.

Regulator-Target gene*	Changes in expression levels of		
	Regulator†	Target Gene†	Control (GAPDH) ‡
GPHN(p)-RALB	-72.6±4.6	40.7±8.7	14.7±12.0
GPHN(a)-RALB	-75.3±3.1	58.2±3.7	9.0±24.2
GPHN(b)-RALB	-88.9±1.0	59.8±15.9	20.9±7.3

* All experiments were based on independent siRNA knockdown of 2 samples.

† Expression levels of the regulators and target genes changed significantly ($P<0.05$, t-test) compared to baseline (without siRNA knockdown).

‡ Expression level of a control, *GAPDH*, did not change significantly ($P>0.05$) upon siRNA knockdown of the regulators.

GPHN p   a pool of 4 siRNA was used (sequences in Supplementary Table 5)
GPHN a   CCAUUGACCUUUUACGUGA
GPHN b   CGAUUUGGAUAAAAGUUGA
