# Supplementary material for: Polymorphic Cis- and Trans-Regulation of Human Gene Expression
Source: PLoS Biol. 2010 Sep 14;8(9):e1000480. doi: 10.1371/journal.pbio.1000480 (PMC2939022; doi:10.1371/journal.pbio.1000480)
Supplement: Table S4 — Primer sequences for qRT-PCR (gene knockdown experiment). (0.04 MB PDF) [file pbio.1000480.s007.pdf]

Supplementary Table 4. Sequences of primers used in RT-PCR to assess expression of regulators and target genes following knockdowns.

Primer Name	Sequence (5’ to 3’)
AIG1-F	TACGGAGGGAGCTGGAAAT
AIG1-R	CTTGAGCTGCCTCTCTTGCT
ACTB-F	TGACCCAGATCATGTTTGAGA
ACTB-R	AGAGGCGTACAGGGATAGCA
BLM-F	ACTTTTGGATTGTGGGAACG
BLM-R	AGGGCCATCAAGTGAATCAG
CLTA-F	CCATCCTTGCTACAGCCTAGA
CLTA-R	CTTTGGCCTGCTTGCTAGAC
FAM120B-F	GGAAGACGAGTGATTTCATGC
FAM120B-R	CTGCCTTCAGGTTGTGGAAT
GALNTL4-F	ATCAAAGTCGTGCGTCACAG
GALNTL4-R	GATGCGGGTGAGTACAGGTT
GAPDH-F	CTCATTTCCTGGTATGACAACG
GAPDH-R	TTACTCCTTGGAGGCCATGT
GCA-F	CATCATGAGTTGCGTCAAGC
GCA-R	TCGAAGCTTCACACAGCAAG
GPHN-F	AACCACTACCTTGGGCACAG
GPHN-R	ACCATGACATCCACCACCTC
HMGCS1-F	ATATGGTTCCCTTGCATCTGTT
HMGCS1-R	CCGGTGTAGCATCTTGTGTG
HSP90AA1-F	TGAACTGGCGGAAGATAAAGA
HSP90AA1-R	CTCATCACCAGAGGCAGATG
HSP90AB1-F	CTGGACGTTCCTTCTTCAGC
HSP90AB1-R	GCTGCTGCGCTATCATACCT
ITGB4BP-F	CCAGGATGAGCTGTCCTCTC
ITGB4BP-R	CAGCTCTGTGCTGGTTGTGT
ITPR2-F	CCTTTTCAAGGTGTGCCCTA
ITPRR2-R	TTTGTTTTTGTTCCAGTTCTGC
KHDRBS3-F	TGGGAAAGGTTCCATGAGAG
KHDRBS3-R	ATGTCCCATCCTGGCATAAG
MEF2A-F	AAGCATGCTCTCTCCACCTC
MEF2A-R	ACTGCTTCCAGCTCCATTTG
MRLC2-F	CAGAGATGGCTTCATCGACA
MRLC2-R	TCACCAAACATGGTCAGGAA
NUSAP1-F	AGGTGCAAGACTGTCCGTGT
NUSAP1-R	TCGTCTGGTGGAGAAGGAAC
PARVA-F	GCAGGAGGAGGGAATGAAC
PARVA-R	TCTTGAAGCTTGGGGTCACT
PSAP-F	CATGCTGCACCTCTGCTCT
PSAP-R	ATCTCCTGCTTGGTGCTGTT
PTPRG-F	GCTGTGGATGTTTTCCAGGT
PTPRG-R	TCATGGGACCATTTCCATTT
RALB-F	AAGAGTGGGGCGTGCAGTA
RALB-R	GCTGCTTTTCTTGCCATTCT
SLC25A11-F	GCCCTCACCAGTATCCTGAA
SLC25A11-R	AGTACCATCAGCCCCAGTCA
TMEM50A-F	CTGTGGCCGTTTTGTTTTCT

TMEM50A-R	CTGAGCATCTCAAGCCCTCT
TTC5-F	CAGGCTAAGTTGGCTGTTCA
TTC5-R	CTTGGGCATAGGCACTGAG
SSR1-F	GGCAATGTATTCCAAGATGC
SSR1-R	AGGAGTTGATGAAGGCCAAC
STK24- R	CCACGAAGGTGTTCCTTTTG
STK24-F	TCCATTCGGAGAAGAAAATCC
VGLL4-F	CTCGCACTGACCAAGAACAG
VGLL4-R	TGCGAGAGGTTGCAGTTG
