# Supplementary material for: Polymorphic Cis- and Trans-Regulation of Human Gene Expression
Source: PLoS Biol. 2010 Sep 14;8(9):e1000480. doi: 10.1371/journal.pbio.1000480 (PMC2939022; doi:10.1371/journal.pbio.1000480)
Supplement: Table S5 — siRNA sequences (gene knockdown experiment). (0.05 MB PDF) [file pbio.1000480.s008.pdf]

Supplementary Table 5. Sequences of siRNAs in knockdown experiments.

Gene Symbol	Genebank ID	siRNA ID	Location	Sequence
AIG1	NM_016108	A-021120-13	3'UTR/ORF	CCUUUAUAUUAAUCGAGAU
AIG1	NM_016108	A-021120-14	3'UTR/ORF	GCAGUGUUCUGGAUCAUUU
AIG1	NM_016108	A-021120-15	3'UTR/ORF	GGCUUUUGUGUAUUUAAUC
AIG1	NM_016108	A-021120-16	3'UTR/ORF	CUGUUGGCUAUAUAUUAUG
BLM	NM_000057	A-007287-14	ORF	GGAUGACUCAGAAUGGUUA
BLM	NM_000057	A-007287-15	ORF	GUCCAUUACUUCAAUAUUU
BLM	NM_000057	A-007287-16	ORF	GACAUAAUCUGAAAUACUA
BLM	NM_000057	A-007287-17	ORF	CCCAUAUGAUUCAGGGAUA
CLTA	NM_001833	A-004002-13	3'-UTR	GGAUUAAUUAUGUUGAGUU
CLTA	NM_001833	A-004002-14	3'-UTR	CUUCUGUGCAUUAUUCUGA
CLTA	NM_001833	A-004002-15	3'-UTR	UUAGAGUUGUUCAUUGUU
CLTA	NM_001833	A-004002-16	ORF	UGUUGAUGGAGUAAUGAAU
FAM120B	NM_032448	A-014898-13	ORF	GCAAGAAGUUUUAAUACGG
FAM120B	NM_032448	A-014898-14	ORF	GCUUUAGGUACAAAUGCUU
FAM120B	NM_032448	A-014898-15	ORF	GCCUAGUUUGAAAAUAUUA
FAM120B	NM_032448	A-014898-16	ORF	GCAACAGAUUUUGAAUUUA
GALNTL4	NM_198516	A-031763-13	3'UTR/ORF	UCUGGAUGGAUGAAUUUAA
GALNTL4	NM_198516	A-031763-14	3'UTR/ORF	GUAUGAUAUCAAACUAUUU
GALNTL4	NM_198516	A-031763-15	3'UTR/ORF	UCAUGGAGUUUAUAUGAAAG
GALNTL4	NM_198516	A-031763-16	3'UTR/ORF	CAGGCUUCAUCAAAGUCGU
GPHN	NM_001024218	A-015811-13	ORF	CCAUUGACCUUUUACGUGA
GPHN	NM_001024218	A-015811-14	ORF	CGAUCAACUUGGGUAUUGU
GPHN	NM_001024218	A-015811-15	ORF	CCAUCAUUUCUCGUGGUGU
GPHN	NM_001024218	A-015811-16	3'-UTR	CGAUUUGGAUAAAAGUUGA
HSP90AB1	NM_007355	A-005187-14	ORF	CGAUUAGGUUAGGAGUUCA
HSP90AB1	NM_007355	A-005187-15	ORF	GCUUCGAGGUGGUAUAUAU
HSP90AB1	NM_007355	A-005187-16	3'-UTR	GCAGUAAACUAAGGGUGUC
HSP90AB1	NM_007355	A-005187-17	ORF	CCAUCACCCUUUAUUUGGA
ITGB4BP	NM_181467	A-010096-14	3'-UTR	GGAUCUAUCAUUACUGCAA
ITGB4BP	NM_181467	A-010096-15	ORF	CAGAUGUGCUCAAGGUGGA
ITGB4BP	NM_181467	A-010096-16	3'-UTR	GAGUCACCUUCCAAGUUGU
ITGB4BP	NM_181467	A-010096-17	3'-UTR	CCAUUAAAGUGCAGUUCCC
ITPR2	NM_002223	A-006208-13	ORF	CUCUUCUGCUAAAUCGAUA
ITPR2	NM_002223	A-006208-14	ORF	GGACGUUGUUAGAUUAUUU
ITPR2	NM_002223	A-006208-15	ORF	GUGUCAGACUGGAUUAUAG
ITPR2	NM_002223	A-006208-16	ORF	CUUUCAUHUCHAAHUUGAU
MEF2A	NM_005587	A-009362-13	ORF	CCUACAACACUGAUUAUUC
MEF2A	NM_005587	A-009362-14	ORF	CCCUGAUACUUCAUAUGUG
MEF2A	NM_005587	A-009362-15	ORF	CUAUGAACUUAGUGUGCUC
MEF2A	NM_005587	A-009362-16	ORF	UUUGUAAACUCAAGAGCUU
PSAP	NM_002778	A-003694-13	ORF	CCAGUAUUCUGAAAUUGCU
PSAP	NM_002778	A-003694-14	ORF	CCAGCAAAGUCUGAUGUUU
PSAP	NM_002778	A-003694-15	ORF	UUCGUGUGCUUGAAAAUUG
PSAP	NM_002778	A-003694-16	ORF	GCUUCAUGCAAGGAGAUAG
TTC5	NM_138376	A-015535-13	ORF	GGCACAAGUUCUAAUGCUA
TTC5	NM_138376	A-015535-14	ORF	CUUGGGAAUUCAUAUCUUU
TTC5	NM_138376	A-015535-15	ORF	GCAACAACUUCUGGAAUUC
TTC5	NM_138376	A-015535-16	ORF	UGAUGGUGUACAAUAUAGU

VGLL4	NM_014667	A-023447-13	3’-UTR	CCAUGUUGUUGGGAUUUGU
VGLL4	NM_014667	A-023447-14	3’-UTR	GCCUUAAGAACAAUAAUAA
VGLL4	NM_014667	A-023447-15	3’-UTR	CUGGUUUCUAUUAAUUAUA
VGLL4	NM_014667	A-023447-16	3’-UTR	GUGUAAAUCUGUAAUAUAC
