# Supplementary material for: Polymorphic Cis- and Trans-Regulation of Human Gene Expression
Source: PLoS Biol. 2010 Sep 14;8(9):e1000480. doi: 10.1371/journal.pbio.1000480 (PMC2939022; doi:10.1371/journal.pbio.1000480)
Supplement: Table S6 — Primer sequences for qRT-PCR (insulin treatment). (0.03 MB DOC) [file pbio.1000480.s009.doc]

Supplementary Table 6. Primer sequences used in RT-PCR to assess the effect of insulin treatment on target genes of INSR.

| Primer Name | Sequence (5’ to 3’) |
| --- | --- |
| INSR-F | TCAGAATGTGACGGAGTTCG |
| INSR-R | CCCTTTGAGGCAATAATCCA |
| ADD3-F | GAGGGAGGGGAAACACAAAG |
| ADD3-R | GGGAGGAGGAGGAGTGGTAA |
| ATIC-F | CGGCGACTACTGCCAACC |
| ATIC-R | CCAGGTCCAGAGTTTCCAGA |
| PSMD-F | ACTCGCCTTGCAGGAGATAA |
| PSMD-R | CAATGTACGCCACACCACTC |
| ARNT-F | GGAAGCTGGAAGAGTTGAA |
| ARNT-R | AGTGCAATGCAGTTCTGCTG |
