# Supplementary material for: Polymorphic Cis- and Trans-Regulation of Human Gene Expression
Source: PLoS Biol. 2010 Sep 14;8(9):e1000480. doi: 10.1371/journal.pbio.1000480 (PMC2939022; doi:10.1371/journal.pbio.1000480)
Supplement: Table S7 — Regulator–target gene pairs that are found to interact physically by Hi-C. (0.05 MB PDF) [file pbio.1000480.s010.pdf]

Supplementary Table 7. Regulator-Target Gene Pairs Found to Interact Physically in HiC (Lieberman-Aiden et al, 2010).

Regulator	Target	T-value	SNP (QTDT)	P-value (QTDT)	SNP	association (P-value)	Hi-C coordinate (regulator)	Hi-C coordinate (target)
ATRN	*NFIB*	5.7	rs151507	0.01	rs151511	N.S.	chr20:3566294	chr9:14180554
USO1	*WDR13*	5.64	rs324734	0.0037	rs7691385	N.S.	chr4:76912713	chrX:48351440
CTNNBIP1	*DDX58*	5.63	rs935073	0.0009	rs935073	N.S.	chr1:9873467	chr9:32472537
ROBO1	*ATF6*	5.38	rs1507417	0.02	rs3821597	0.0059	chr3:79217935	chr1:160093368
TMEM45A	*KCNMA1*	5.06	rs6799992	0.01	rs10936221	N.S.	chr3:101764442	chr10:78646999
KCNMA1	*HMGCS1*	5.04	rs11002137	0.0058	rs10824547	0.01	chr10:78782737	chr5:43334692
KCNMA1	*HMGCS1*	5.04	rs11002137	0.0058	rs10824547	0.01	chr10:78778388	chr5:43334974
KCNQ5	*ETV6*	5.04	rs16883476	0.0002	rs16882712	N.S.	chr6:73892628	chr12:11800513
KCNQ5	*ETV6*	5.04	rs16883476	0.0002	rs16882712	N.S.	chr6:73522933	chr12:11841656
PRKCE	*LIG4*	4.99	rs2711295	0.0004	rs4953279	0.0077	chr2:45893756	chr13:107664041
PHLPP	*PFKL*	4.88	rs2053600	0.03	rs10153412	N.S.	chr18:58718878	chr21:44555603
ROBO1	*VRK2*	4.83	rs9838937	0.0012	rs9828140	0.02	chr3:79422794	chr2:58139091
ROBO1	*VRK2*	4.83	rs9838937	0.0012	rs9828140	0.02	chr3:79020607	chr2:58189813
WWOX	*IMPA2*	4.82	rs11150104	0.0018	rs9932697	0.01	chr16:76861753	chr18:11973686
DIS3L2	*GLTSCR2*	4.82	rs3100608	0.01	rs16828779	N.S.	chr2:232554756	chr19:52951146
SDCCAG8	*ACBD3*	4.82	rs11800122	0.0078	rs2802722	N.S.	chr1:241732306	chr1:224420181
SDCCAG8	*ACBD3*	4.82	rs11800122	0.0078	rs2802722	N.S.	chr1:241640785	chr1:224442930
SDCCAG8	*ACBD3*	4.82	rs11800122	0.0078	rs2802722	N.S.	chr1:241614818	chr1:224416160
SDCCAG8	*NDEL1*	4.81	rs10803140	0.0088	rs3006917	N.S.	chr1:241615074	chr17:8316669
SMYD3	*COX4NB*	4.75	rs2105158	0.0033	rs4445429	0.005	chr1:244566879	chr16:84369002
FAM120B	*KHDRBS3*	4.72	rs910424	0.00004	rs1022615	0.0022	chr6:170532078	chr8:136625771
ZFYVE20	*PBX3*	4.72	rs9845081	0.0026	rs9845081	N.S.	chr3:15088788	chr9:127660328
TRERF1	*STK24*	4.71	rs489743	0.0076	rs2025429	N.S.	chr6:42413750	chr13:97949109
STX2	*PDK1*	4.69	rs11061149	0.05	rs11061149	N.S.	chr12:129850093	chr2:173137207
C20orf43	*CAPZA2*	4.67	rs4811702	0.01	rs3787395	0.02	chr20:54494255	chr7:116326936
SFMBT2	*MGMT*	4.67	rs4748900	0.03	rs638145	0.02	chr10:7388877	chr10:131393555
JMJD2C	*CLTA*	4.66	rs12380879	0.0036	rs10976063	0.0029	chr9:7125314	chr9:36197153
NUFIP2	*ZDHHC13*	4.65	rs8080081	0.01	rs16964963	N.S.	chr17:24622455	chr11:19140369
C20orf94	*SKAP1*	4.65	rs637688	0.01	rs16991827	N.S.	chr20:10541903	chr17:43602188
C20orf94	*SKAP1*	4.65	rs637688	0.01	rs16991827	N.S.	chr20:10522265	chr17:43658852
SCHIP1	*PIK3CB*	4.63	rs4680509	0.02	rs1675496	0.000003	chr3:160645011	chr3:139954130
SCHIP1	*PIK3CB*	4.63	rs4680509	0.02	rs1675496	0.000003	chr3:161094075	chr3:139890662
LRRC8D	*PSMD14*	4.61	rs17130931	0.01	rs1938032	0.03	chr1:90120587	chr2:161885030
HCCA2	*SLC25A14*	4.59	rs17832809	0.03	rs17834326	0.02	chr11:1600413	chrX:129306975
ESCO2	*BIN3*	4.55	rs4732754	0.0017	rs4732754	N.S.	chr8:27692067	chr8:22579065
RORA	*GTF2F2*	4.54	rs4775313	0.0085	rs11632352	0.0011	chr15:58726758	chr13:44666011
RORA	*GTF2F2*	4.54	rs4775313	0.0085	rs11632352	0.0011	chr15:58776756	chr13:44714031
TCF4	*TOPBP1*	4.53	rs1261134	0.0335	rs9636107	0.04	chr18:51115285	chr3:134824464
UXS1	*MNAT1*	4.44	rs2167531	0.0284	rs17032398	N.S.	chr2:106178704	chr14:60277086
RFTN1	*NPAT*	4.44	rs689953	0.0004	rs508808	0.03	chr3:16497316	chr11:107551792
SNX29	*ARFGEF2*	4.43	rs13336249	0.0012	rs350272	0.02	chr16:12574694	chr20:46973141
FAM173B	*PDE3B*	4.43	rs11742483	0.0275	rs4702678	N.S.	chr5:10274967	chr11:14840807
ZFR	*PCTP*	4.4	rs2963980	0.0006	rs13155643	N.S.	chr5:32444043	chr17:51204238
MRPL44	*SP140*	4.38	rs1025734	0.0071	rs1025734	N.S.	chr2:224527572	chr2:230819878
MRPL44	*SP140*	4.38	rs1025734	0.0071	rs1025734	N.S.	chr2:224527631	chr2:230837973
SFRS12IP1	*NRF1*	4.38	rs275818	0.05	rs1824353	0.0004	chr5:64095824	chr7:129088802
AEBP2	*CAPNS1*	4.38	rs7297431	0.0033	rs4963538	0.03	chr12:19551760	chr19:41329623
ETNK1	*PCMT1*	4.38	rs4963842	0.02	rs17426308	N.S.	chr12:22669012	chr6:150140666
FHIT	*MTMR2*	4.37	rs10510833	0.0026	rs17364716	0.0026	chr3:61031047	chr11:95206501
COMMD10	*XRCC4*	4.36	rs10042163	0.0007	rs250331	N.S.	chr5:115637321	chr5:82594176
CACNA1E	*KIAA0859*	4.36	rs17495655	0.01	rs10489641	0.02	chr1:179831416	chr1:170014674
CAMK4	*ARS2*	4.36	rs10491334	0.03	rs434256	N.S.	chr5:110650815	chr7:100319355
COMMD10	*XRCC4*	4.36	rs10042163	0.0007	rs250331	N.S.	chr5:115479881	chr5:82464495
MACROD2	*MGEA5*	4.34	rs17775664	0.0013	rs4141734	0.0005	chr20:14927669	chr10:103563144
AIG1	*TMEM50A*	4.28	rs9403484	0.0034	rs9390075	N.S.	chr6:143639643	chr1:25548869
CACNA1E	*SNX7*	4.27	rs2877712	0.02	rs199949	0.01	chr1:179818091	chr1:98972537
STXBP6	*INHBC*	4.24	rs8019575	0.0057	rs9323564	N.S.	ch14:24453027	chr12:56117910
VPS41	*IQGAP2*	4.23	rs17171473	0.0012	rs17767818	N.S.	chr7:38823282	chr5:75787559
ELOVL6	*UBE2D3*	4.22	rs34392886	0.007	rs34415611	0.04	chr4:111200009	chr4:103968808
ELOVL6	*UBE2D3*	4.22	rs34392886	0.007	rs34415611	0.04	chr4:111301411	chr4:103968914
ACSS2	*SIL1*	4.21	rs6088650	0.0043	rs1013677	N.S.	chr20:32973223	chr5:138477927
EFNA5	*DAPK1*	4.19	rs17534270	0.03	rs29543	0.0007	chr5:106902938	chr9:89360859
WWP1	*ANK2*	4.18	rs10093238	0.01	rs6996052	N.S.	chr8:87443641	chr4:114248967
NPHP4	*CYP4F3*	4.16	rs2312464	0.02	rs12737596	N.S.	chr1:5868436	chr19:16054014
STRN3	*SCMH1*	4.16	rs10444696	0.04	rs10129393	N.S.	chr14:30479942	chr1:41264023
GPHN	*RALB*	4.16	rs8022657	0.0025	rs7154017	0.02	chr14:66403677	chr2:120766519
GALNTL4	*PTPRG*	4.15	rs9943596	0.0009	rs10765861	0.02	chr11:11407485	chr3:62126269
VGLL4	*UBE4B*	4.14	rs2574717	0.0044	rs13075818	0.04	chr3:11673423	chr1:10016836
DOCK8	*TM6SF1*	4.14	rs17721320	0.0231	rs10967642	0.03	chr9:317258	chr15:81579701
GFOD1	*DBT*	4.13	rs536998	0.0038	rs6936420	N.S.	chr6:13556895	chr1:100437973
ASTN2	*BUB1B*	4.13	rs10817960	0.0086	rs7023773	0.0018	chr9:118294444	chr15:38282392
ASTN2	*BUB1B*	4.13	rs10817960	0.0086	rs7023773	0.0018	chr9:118868352	chr15:38272245
DENND1A	*CKS1B*	4.12	rs7026921	0.0052	rs7026808	0.0069	chr9:125477094	chr1:153218248
INSR	*CCL5*	4.05	rs890862	0.04	rs16994200	N.S.	chr19:7159737	chr17:31223009
FGD2	*TMEM187*	4.04	rs831483	0.0064	rs4714031	N.S.	chr6:37109432	chrX:152899132
